# Community-Based Delivery and Administration of SARS-CoV-2 Antigen Rapid Diagnostic Tests: An Operational Research Study in Marketplaces in Malawi and Zambia

**DOI:** 10.4269/ajtmh.23-0785

**Published:** 2024-10-01

**Authors:** Fiona Gambanga, Lindiwe Nchimunya, Joseph Makondesa, Chancy Chavula, Namwaka Mulenga, Tamara Mwenifumbo, Francis Chitanda, Jonathan Mtaula, Yucheng Tsai, Joseph Bitilinyu-Bangoh, Andrews Gunda, Aaron Shibemba, Powell Choonga, Shaukat Khan, Trevor Peter

**Affiliations:** ^1^ Clinton Health Access Initiative, Boston, Massachusetts;; ^2^ Ministry of Health, HTSS-Diagnostics, Lilongwe Central Region, Malawi;; ^3^ Ministry of Health, Laboratory Services, Lusaka, Zambia;; ^4^ FIND, Geneva, Switzerland

## Abstract

To expand access to testing beyond public health facilities and to strengthen surveillance efforts for COVID-19, community testing using COVID-19 antigen-based rapid diagnostic tests (Ag-RDTs) was identified as a major area of focus in Malawi and Zambia. This research aimed to gather evidence on the feasibility and acceptability of community testing in marketplaces. A cross-sectional study with a mixed-methods design was conducted in marketplaces in Malawi and Zambia to understand operational considerations for the implementation of Ag-RDTs for SARS-CoV-2 in a community setting. Programmatic data were collected prospectively as individuals were tested from June to September 2022. COVID-19 testing was done using Abbott Panbio nasal swab test kits. Semi-qualitative questionnaires were administered to individuals who tested, healthcare workers, and site-based personnel. Data were collected electronically via the SurveyCTO platform and analyzed using STATA. In Malawi, 2,348 participants were tested, and in Zambia, 1,723 people were tested for COVID-19. In Zambia, participants were 46% female, with a median age of 28 years, whereas in Malawi, participants were 69% female, with a median age of 37 years. In Malawi, 78 positive cases were reported (3.3% positivity rate), and in Zambia 10 positive cases were reported (0.5% positivity rate). In Zambia, 99% of 300 participants and in Malawi, 92% of 1,158 testers found the market testing experience and sample collection acceptable. Community testing is a feasible and acceptable intervention to increase testing access in Malawi and Zambia, especially when coupled with community awareness campaigns and mobilization.

## INTRODUCTION

Zambia and Malawi recorded their first coronavirus disease 2019 (COVID-19) cases in March and April 2020, respectively. Since then, several system-strengthening activities have been done to increase access to testing and support interventions related to testing in the two countries. COVID-19 testing in the two countries has been largely limited to facility-based settings and targeted high-risk populations including symptomatic or contacts of index cases, which have limited the testing average to about 500 tests a day in Malawi and a range between 800 and 1,000 a day in Zambia, which is well below the WHO recommendations of one test per 1,000 population per week.^1^ In addition, testing coverage remained suboptimal owing to limited access to testing centers and long queues in facility-based centers, discouraging people from accessing testing.

Severe acute respiratory syndrome coronavirus 2 (SARS-CoV-2) antigen-detection diagnostic tests offer a faster and cheaper alternative in diagnosing active COVID-19 infection in comparison to polymerase chain reaction or nucleic acid amplification tests. They are designed to directly detect SARS-CoV-2 proteins produced by replicating the virus in respiratory secretions. Antigen tests have been developed for use either at centralized laboratories or as point-of-care tests. Antigen rapid diagnostic tests (Ag-RDTs) can either be conducted using device-free lateral flow assays or assays that are read by a reader/device at the test site, and they do not require laboratory infrastructure and can be conducted by healthcare workers or other trained personnel. Both Ag-RDT types can provide results within 15 minutes.^2^ Given the infectiousness of SARS-CoV-2 in both symptomatic and asymptomatic individuals, a fast turnaround of results (same day) is critical to change the rapid isolation of cases as well as the tracing of contacts. It also facilitates more routine testing in places such as health facilities, schools, marketplaces, pharmacies, workplaces, and prisons. Furthermore, nonreliance on central laboratories allows for increased decentralization of SARS-CoV-2 testing; this increases the volume and equity of testing across a country and allows the country to detect and contain outbreaks as they occur.

To maximize testing coverage and gather evidence for surveillance purposes in partnership with district health offices, this study delivered Ag-RDT testing in community settings within highly trafficked areas in markets in Zambia and Malawi. The study was conducted in these two countries because of the similarities in the challenges experienced in the COVID-19 testing landscape and the need to improvise strategies to scale up testing coverage and surveillance.

The aim of the operational research was to answer the following research questions: 1) What are the operational considerations for introducing Ag-RDT in a community setting? 2) What is the feasibility and acceptability of Ag-RDT implementation?

Feasibility and acceptability of Ag-RDT in this study was defined as the demand for the test, demonstrated by community participants’ willingness to be tested. The study also gathered operational considerations from the health providers’ perspective and patient perspective on community-based testing as provided at the time from various qualitative questions.

The operational research study had four main objectives:

1. Describe the implementation process and challenges associated with the deployment and administration of Ag-RDT.
2. Measure the extent to which Ag-RDT was implemented for surveillance in the marketplace.
3. Assess community health workers and individual perspectives on the feasibility and acceptability of Ag-RDT in the marketplace.
4. Measure the success of quarantining after being tested in a community setting (marketplace).

## MATERIALS AND METHODS

### Study design.

This was a cross-sectional study with a mixed-methods design for understanding in more detail operational considerations for implementation of Ag-RDTs for SARS-CoV-2 in community settings in Malawi and Zambia. The programmatic data were collected prospectively as individuals were tested. Implementation of COVID-19 testing was done using the Abbott Panbio Nasal swab test kit (Abbott Park, IL), as it has received emergency use authorization from relevant regulatory agencies and is nationally registered for clinical use in both countries. A criterion was set to meet a threshold of 1,200 people on the upper limit sample size accepting a COVID-19 test in the market setup and 800 on the lower side in Zambia, whereas in Malawi, the criteria for acceptance was an average of 20 people tested per day.

The study was rolled out in one of the largest markets in Lusaka province in collaboration with local town council and market authorities. Their role was to introduce the study and testing personnel in the market and drum up support for the study among traders and patrons of the market. The market services three surrounding neighborhoods and communities and is visited by more than 800 people a day. The study sites were set up in four different locations of the market, and COVID-19 Ag-RDT was offered to the study population, which included traders and buyers in the market. In Zambia, the study was conducted weekly over 14 test dates across 3 months (June, July, and August 2022).

In Malawi, the study was rolled out in four purposively selected markets in two districts of Lilongwe and Blantyre. Purposive sampling was used based on the size of the market. Daily testing services were provided at the testing sites for 74 days between July and September 2022. In Malawi, the study estimated that 200 people would access each market each day.

The study targeted the population in the markets, which is considered to be at the highest risk of acquiring the infection and also has the potential to become a super-spreader. In addition to the vendors, the testing sites also offered voluntary testing to individuals who were accessing the markets. The study engaged surveillance teams from the Ministry of Health (MOH) to conduct community awareness programs such as health education in the market through audio and visual announcers informing citizens about the community testing sites and facilitating the capture of testing in MOH-approved registers.

In both countries, the study was composed of clinical evaluation and administration of semi-structured surveys. For the clinical evaluation component, the participant inclusion criteria were any individual who accessed the testing site, was 18 years old and above, and received an Ag-RDT.

### Data collection.

Data were collected prospectively via SurveyCTO at the testing sites by using a set of questionnaires and MOH-approved COVID-19 registers for all people tested. The questionnaires included individual questionnaires for people enrolled in the research, a testing site information questionnaire, a healthcare worker questionnaire, and a post-test survey questionnaire.

In Zambia, every fifth individual who got tested was eligible to complete the individual questionnaire. The test procedure involved having participants sit comfortably in a designated testing area, and the test was administered using a nasal swab RDT, which is less invasive than the nasopharyngeal swab. In Malawi, the selection of respondents given the individual questionnaire was random. Individuals who tested positive and consented to be followed were contacted by phone to determine their satisfaction with and completion of self-quarantine. Site staff collected the testing site information sheet and health worker questionnaire to assess the implementation of community testing. For all questionnaires, both quantitative and qualitative outcomes were collected, including information about whether they had ever been tested before, why they chose to get tested, and how acceptable they found the testing experience.

For the semi-structured survey component, the study used three questionnaires: one for healthcare workers and testing staff involved in administering the Ag-RDTs, another for individuals who received a SARS-CoV-2 diagnosis, were 18 years or older, were willing and able to provide consent, and had received an Ag-RDT. The last questionnaire was a follow-up survey for individuals who tested positive and consented to phone call follow-up 10–14 days after receiving their positive result based on the national COVID-19 testing guidelines for the two countries.

The healthcare worker questionnaire asked detailed questions on any site-based adaptations as well as challenges and facilitators of implementing testing of Ag-RDT in general for each of the use cases. The individual questionnaires explored the level of individual satisfaction with Ag-RDT and the delivery of services; in addition, for a subset of individuals who tested positive, there were questions asking for their experiences with self-quarantining.

All healthcare workers at the testing site who were involved in the administration of Ag-RDT testing and/or clinical management of COVID-19 were invited to participate in the surveys. The study involved various healthcare worker cadres, including laboratory technicians, clinical officers, nurses, and health surveillance assistants. All of these cadres received training in COVID-19 testing using the Panbio RDT kit. To ensure quality control and assurance, in addition to following the WHO guidelines for RDT COVID-19 testing, Panbio RDT kit was used because it is prequalified by the WHO, ensuring its reliability and accuracy. Control tests were also conducted at the beginning of each day at the test sites to validate the accuracy of the results.

**Figure 1.**
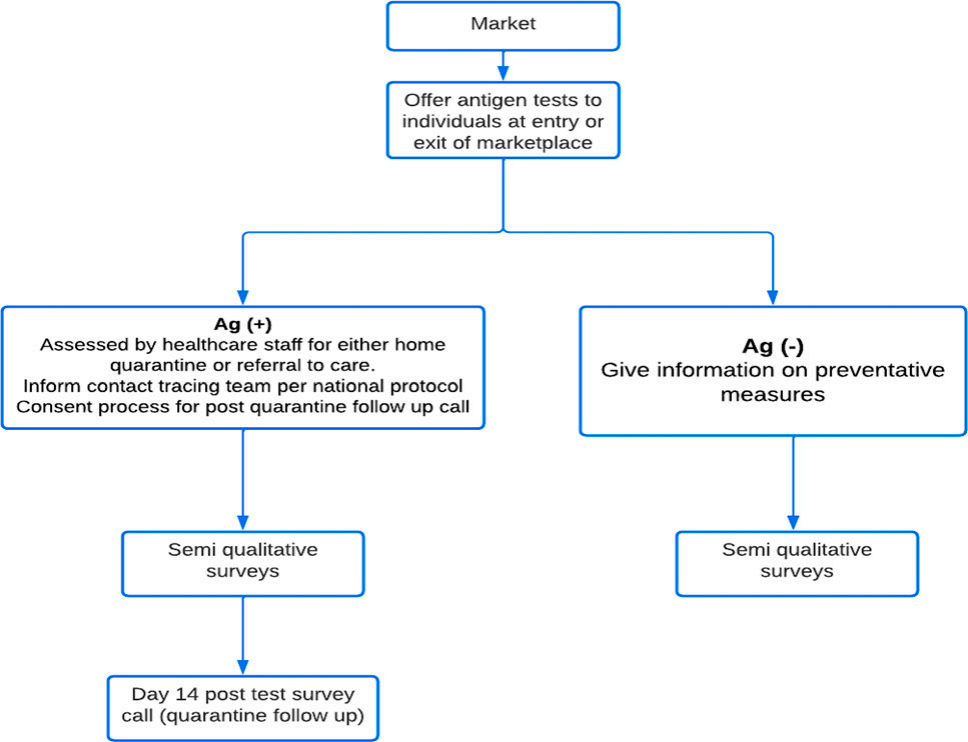
Study flowchart. Ag = antigen.

For individual surveys, the individuals were recruited after they received their test, with healthcare workers inviting individuals to participate in the survey and providing study information in the participants’ preferred language (English, Chichewa, or Bemba). The healthcare workers interviewing the participants received adequate training on the survey process and interviewing skills. Individuals who agreed to participate were asked to provide written informed consent and were surveyed after consenting, with each interview lasting between 10 and 15 minutes; responses were written on electronic-based questionnaires. In addition, all individuals who tested positive were invited to participate in follow-up surveys after 10–14 days of quarantine to determine their satisfaction and experience with quarantine (see Figure 1).

All electronic data were stored in the SurveyCTO database with data security protection by a password. The paper-based register was taken back to the district health offices in each district after each day of use and were kept in a locked office.

Quantitative data from the individual questionnaire, MOH-approved register, healthcare worker questionnaire, and post-test survey were analyzed using STATA (College Station, TX) and descriptively summarized in Microsoft Excel (Redmond, WA). Qualitative data from the open-ended questions from the surveys and questionnaires were analyzed using thematic analysis by creating codes and summarizing the number of entries per specific code.

The study obtained ethics approval from the Clinton Health Access Initiative Scientific and Ethics Research Committee. In addition, we obtained ethics clearance from the National Health Sciences Research Committee in Malawi (approval number 2900) and ERES Converge Institutional Review Board in Zambia (Ref. No. 2022-Mar-017).

## RESULTS

The study set out to evaluate the feasibility and acceptability of community-based delivery and administration of SARS-CoV-2 Ag-RDT in Malawi and Zambia. The study population consisted of a combined total of 4,071 participants who voluntarily got tested for COVID-19 at Chichiri Shopping Mall, Game Complex, Lunzu and Wakawaka markets in Lilongwe and Blantyre districts in Malawi, and Mtendere Market in Lusaka, Zambia.

### Demographics of COVID-19–testing participants.

At the end of the study testing period, 2,348 and 1,723 participants in Malawi and Zambia, respectively, were tested for COVID-19. In Malawi, 69% of participants were female, and the median age was 27 years (interquartile range [IQR]: 27–46 years) (minimum 2, maximum 99), whereas in Zambia, 46% of participants were female, and the median age was 28 years (IQR: 20–39 years) (min 0, max 87) (see Table 1).

**Table 1:** Summary table of the population tested for SARS-CoV-2 using antigen tests at marketplaces in Malawi and Zambia from May to August 2022

Variables	Malawi, *N* (%)	Zambia, *N* (%)
Total Number of Sites	4	1
Testing Period	74 Test Days across 3 Months	14 Test Days across 3 Months
Total Number of People Tested	2,348	1,723
Median Age	27 Years (IQR: 27–46 years)	28 Years (IQR: 20–39 years)
Sex		
Male	725 (31)	932 (54)
Female	1,622 (69)	791 (46)
Undisclosed	1 (0)	0 (0)
Symptom Status		
Symptomatic	523 (22)	7 (1)
Asymptomatic	1,825 (78)	1,716 (99)
COVID-19 Ag-RDT Test Result		
Positive	78 (3)	10 (1)
Negative	2,270 (97)	1,713 (99)
Invalid	0 (0)	0 (0)

Ag-RDT = antigen-based rapid diagnostic test; COVID-19 = coronavirus disease 2019; IQR = interquartile range; SARS-CoV-2 = severe acute respiratory syndrome coronavirus.

### Number of people who tested positive.

From the total tests conducted, 88 positive cases across the 5 testing sites in the two countries were recorded. Of the total tests conducted in Malawi, 78 positive cases were reported (3.3% positivity rate) and in Zambia, 10 positive cases were reported (1% positivity rate). In Malawi, 70 of the positive cases were identified as index cases, and the remaining eight were contacts, whereas in Zambia, eight cases were index cases and two were contacts (see Table 2).

**Table 2:** Profile of participants who tested positive for SARS-CoV-2 using antigen tests at marketplaces in Malawi and Zambia from May to August 2022

Variables	Malawi, *N* (%)	Zambia, *N* (%)
Total Number of People who Tested Positive	78	10
Sex		
Male	53 (68)	6 (60)
Female	25 (32)	4 (40)
Index Status		
Index Cases	70 (90)	8 (80)
Contact Cases	8 (10)	2 (20)

### Clinical attributes of COVID-19–testing participants in Malawi.

**Figure 2.**
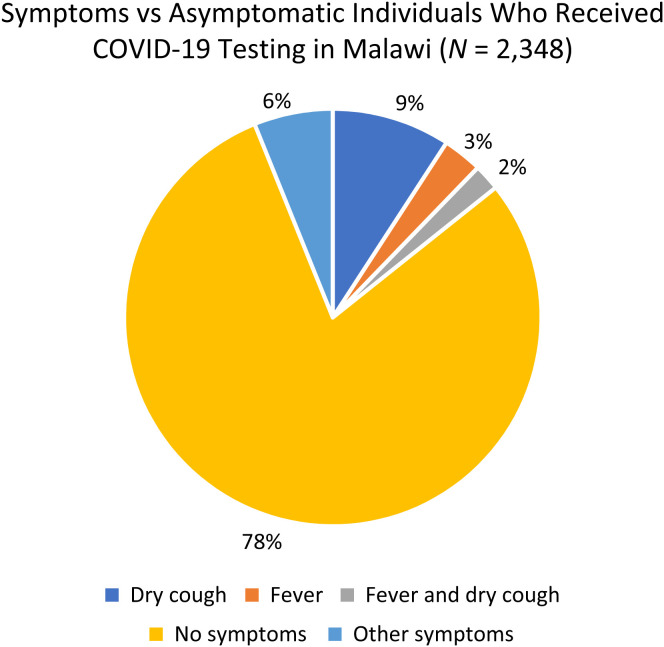
Symptomatic versus asymptomatic participants who received COVID-19 testing in Malawi. COVID-19 = coronavirus disease 2019.

From the tests conducted in Malawi, 78% of clients were asymptomatic (Figure 2). Of the 22% of symptomatic clients, dry cough was the most-often reported symptom at 9%, 3% of the clients had a fever, whereas 2% reported having both dry cough and fever as symptoms. All positive cases were referred to the nearest clinic and advised to self-isolate for 10 days as laid out in the national guidelines for COVID-19 in Malawi. No individuals required hospitalization after being found positive. Figure 2 shows various symptoms among the individuals who received COVID-19 testing. Supplemental Figure 1 shows self reported symptoms among those who tested positive (n=78) in Malawi.

### Clinical attributes of COVID-19–testing participants in Zambia.

In Zambia, 99% of participants had no symptoms, and 1% signed up to get tested because they were in a high-risk group.^†^ Dry cough and congestion were the most common symptom combination (6%) for those who reported symptoms. Of all tested, 1% of the population tested positive (*N* = 10) (see Tables 1 and 2; Supplemental Figure 2).

### Study participants’ survey responses.

**Figure 3.**
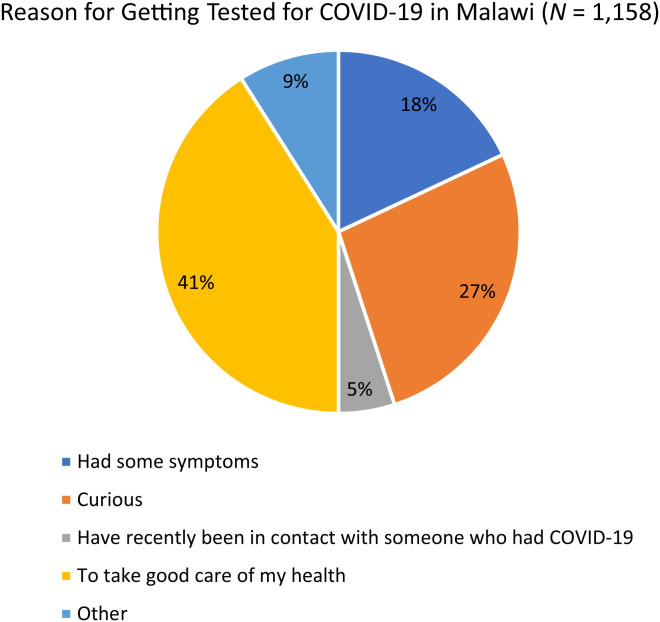
Reason for choosing to get tested for COVID-19 at the marketplace in Malawi. COVID-19 = coronavirus disease 2019.

In Malawi, 1,158 respondents were surveyed, among whom 70% were male (30% female) with a median age of 37 (IQR: 27–46) years, and 3.3% had tested positive. Of the individuals surveyed in Malawi, 53% reported that they had never been tested for COVID-19. Of those previously tested for COVID-19, 70% had been tested at a hospital in Malawi. Regarding the main reason clients chose to get tested in Malawi, 41% reported that they tested for COVID-19 in a community setting because they wanted to take good care of their health, whereas 27% reported that they were curious about community testing and hence chose to get tested (see Figure 3).

In Zambia, 300 respondents were surveyed, among whom 47% were female with a median age of 31 years (IQR: 24–41 years). In Zambia, 59% of the respondents indicated the desire to know their COVID-19 status as the major reason for testing, and 32% cited wanting to take care of themselves and their health. Of the respondents, 49% had previously been tested for COVID-19. Of those who had previously received a test for COVID-19, 70% had received that test at a hospital.

After COVID-19 testing, clients were asked whether they found the sample collection experience acceptable. In Zambia, all respondents found the sample collection experience to be either acceptable (33%) or very acceptable (67%). Similarly, all the respondents found the overall testing experience at the market acceptable (33%) or very acceptable (67%). For Malawi, 92% reported that they found the sample collection process acceptable and less painful than the nasopharyngeal sample collection procedure. Ninety-three percent reported that they found the whole testing experience acceptable, citing that it was fast and very time efficient.

Most of the respondents in Malawi and Zambia (99%) reported that they trusted the test result. Of those who did not trust the test results, reasons included the short turnaround time for the results, general distrust, inconsistencies with the test results, and having all the symptoms of COVID-19 but never testing positive for COVID-19. Most of the respondents also said they would consider future testing (98% for Malawi and 99% for Zambia) and that they would recommend community testing to their family and friends. The main reason among the participants who reported not considering future testing was that the testing was painful and made them feel uncomfortable, and they would prefer another method of collecting samples rather than a nasal swab.

**Figure 4.**
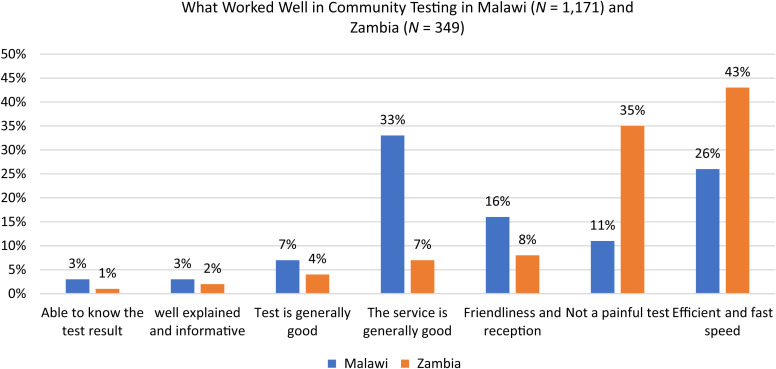
What worked well in community testing in Malawi and Zambia.

Major feedback on what worked well during the testing process included receiving the COVID-19 antigen-based community testing, service efficiency, and the short turnaround time, accounting for 26% in Malawi and 43% in Zambia. At each site, one clinician, one or two data collectors, and one or two testers were deployed, and the team provided the testing service in a smooth flow, from registering individuals for basic information, conducting the tests, providing feedback on the test result, and enrolling the eligible population into the research for additional data collection. In addition to the efficient services, the short turnaround time of the RDT also shortened the waiting time for people getting tested and time to receiving their results. Figure 4 outlines the issues that respondents highlighted as having worked well in community testing in Malawi and Zambia.

**Figure 5.**
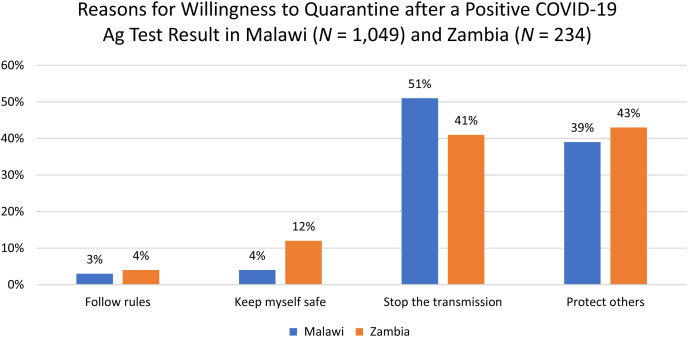
Reasons for willingness to quarantine after a positive COVID-19 Ag test result in Malawi and Zambia. Ag = antigen; COVID-19 = coronavirus disease 2019.

The clients who volunteered to take part in the surveys were asked whether they would complete self-isolation if they tested positive; 90% and 69% of the respondents expressed that they would quarantine if they tested positive in Malawi and Zambia, respectively. The participants were further asked to provide the reasons why they would consider quarantining/isolating after testing positive, and the main reasons given are outlined in Figure 5.

In Malawi, the main reason given among the clients who reported not considering quarantine after testing positive (10%) was that they are breadwinners, and if they quarantine, their households would be affected financially. In Zambia 28% reported that it was either unlikely or highlight unlikely that they would quarantine if they got a positive result, with the majority mentioning that they had to work or go to school (67%). Some respondents also shared that they were unwilling to have their lifestyle changed or disturbed by COVID-19, accounting for 14% of the respondents.

### Healthcare workers’ survey responses.

For the healthcare worker questionnaire, there were 61 respondents (51 in Malawi and 10 in Zambia). The healthcare worker questionnaire helped to assess community healthcare worker and staff perspectives on the acceptability and feasibility of Ag-RDT in a marketplace. All the healthcare workers available at the community testing sites had received training for COVID-19 testing. Healthcare workers were asked to report what worked well in the community testing sites, and the following are some of the responses provided: publicity using health promotion teams from the MOH, which contributed to more community awareness and hence good turnout of clients; good organization among the staff; adequate staffing levels at the community testing sites; no stockouts experienced throughout the study, which prevented clients from going back home without getting tested; and the short turnaround time for the tests conducted (same day within an hour).

In addition to this, in Malawi the healthcare workers reported the following factors as limiting the success of community testing: myths about COVID-19 among community members, leading to lower acceptance rates for testing; lack of awareness among community members on community testing for COVID-19, as people are used to facility-based testing; and absence of vaccination services at the testing site, as some of the people would only visit the site to get a vaccine and would turn back if they saw that there was no vaccination service being offered. Almost all the healthcare workers reported that there should be increased community awareness and sensitization to inform people about community testing and dispel the myths surrounding community testing.

### Post-test follow-up survey responses.

**Figure 6.**
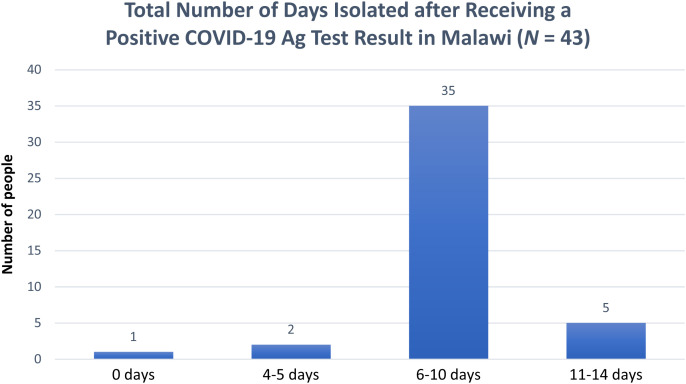
Total number of days isolated after receiving a positive COVID-19 Ag test result in Malawi. Ag = antigen; COVID-19 = coronavirus disease 2019.

**Figure 7.**
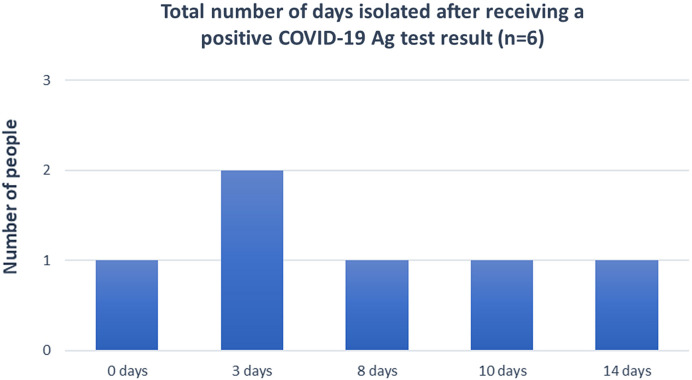
Total number of days isolated after receiving a positive COVID-19 Ag test result in Zambia. Ag = antigen; COVID-19 = coronavirus disease 2019.

Among the 88 individuals who tested positive in the study (78 in Malawi and 10 in Zambia), 49 consented to be followed up for a post-test survey through a telephone poll after 10–14 days from the day they tested positive. In Malawi, 43 respondents consented, whereas in Zambia, six people consented to the post-test survey. Sixty-seven percent of these were males (33% females). From these surveys, 76% of the respondents reported that they isolated for 6–10 days after testing positive, and 12% self-isolated for 11–14 days. Of the 43 respondents in Malawi, 28% said they found self-isolation to be a challenge. Figures 6 and 7 show the total number of days individuals were isolated after receiving a positive test result in Malawi and Zambia, respectively.

Varied reasons were given for why self-isolation was a challenge, and among these were financial problems due to failure to go to work for daily income, failure to associate with people, and being limited from doing piecework. When asked about the preventive measures that those who tested positive took during the self-isolation period, 26% reported that they wore a mask as a preventive measure together with working from home and informing contacts about their COVID-19 status.

## DISCUSSION

This study gave an insight into the acceptability and feasibility of community testing for COVID-19 in a market setting in Zambia and Malawi. The feasibility of community testing appeared realistic given the simple testing procedure in using the RDT for both healthcare workers and clients accessing COVID-19 testing services at community testing sites. The results of our study are in line with those of Omollo et al.,^3^ who studied health workers’ perceptions of the feasibility and acceptability of introducing Ag-RDT for COVID-19 in Kisumu County in Kenya; they found that the workers accepted the use of Ag-RDT because it enabled the strengthening of the existing health system, increased testing capacity, and provided capacity-building opportunities through the training provided to the healthcare workers before conducting testing for COVID-19. The results have also shown that community testing for COVID-19 using Ag-RDT increases testing capacity. During the study period in Malawi, community testing from the four study sites accounted for 21% of the total national antigen tests for COVID-19. This was significant considering there were 357 facility-based testing sites operating across the country at the same time.

In addition, the training provided at the beginning of the study to healthcare workers, offering COVID-19 services and the mentorship and supportive supervision that were offered to the health workers during the implementation of the study, helped to increase their knowledge about the provision of COVID-19 testing services.^4^

The study also found that cooperation with local authorities, including the market cooperatives and clinics, was important for community-based testing interventions. The market cooperatives provided substantial support on site selection, communication with residents, and identifying awareness campaigners who were familiar with the market. By collaborating with district MOH officials and local clinics, the teams in both countries were able to discuss referral and surveillance for positive patients and conduct biomedical waste management.

In addition to this, we noted that advocacy and awareness raising are key in increasing the demand for COVID-19 testing. During the research period, some individuals came up to ask about other services besides COVID-19, for example, integrating COVID-19 testing with vaccination or other rapid tests such as HIV. To motivate people to receive COVID-19 Ag-RDT without any incentives provided, advocacy is key to scaling the availability of free testing services, as well as providing messages on why the testing is important to individuals and the people around them. Advocacy and public awareness help to address misconceptions about the consequences of testing.^5^

Key implementation challenges included the saturation of testing at the study sites, the difficulty of contact tracing, and reduced motivation for receiving tests by the general public. We learned that relocating the testing sites would be helpful to reach different populations. At the time the saturation was observed, the team discussed with the market cooperatives the relocation of three of the four sites to other locations within the market.

### Limitations of the study.

Given that this was an observational instead of a randomized study, we are not able to assess the effectiveness of using COVID-19 rapid antigen tests in marketplace settings. Another limitation was self-selection bias from the participants, given that all tests were voluntary. Lack of data and feedback from individuals who did not wish to be surveyed in terms of understanding or measuring acceptability may have skewed the feedback toward responses from those who opted in. Lastly, external factors were not controllable, such as changes in COVID-19 testing guidelines, the situation of the epidemic, and uncertainty around upcoming waves.

## CONCLUSION

Community testing is a feasible and acceptable intervention to increase testing access in Zambia and Malawi for both clients and healthcare workers, especially when coupled with community awareness and advocacy, education, and mobilization. Integrated services at the community level should also be considered. This strategy provides an additional testing modality for countries to manage future epidemics.

## Supplemental Materials

10.4269/ajtmh.23-0785 Supplemental Materials

## Footnotes

^† ^
High risk includes people over 65 years; people with preexisting conditions such as diabetes, pulmonary conditions including asthma; those living with HIV; and those who are obese or have a weakened immune system.
