# Supplementary material for: Community-Based Delivery and Administration of SARS-CoV-2 Antigen Rapid Diagnostic Tests: An Operational Research Study in Marketplaces in Malawi and Zambia
Source: Am J Trop Med Hyg. 2024 Oct 1;112(4 Suppl):63–70. doi: 10.4269/ajtmh.23-0785 (PMC11965721; doi:10.4269/ajtmh.23-0785)
Supplement: Supplemental Materials [file tpmd230785.SD1.pdf]

Supplementary figures

SUPPLEMENTARY FIGURE 1: SELF-REPORTED SYMPTOMS OF POSITIVE CASES IN MALAWI

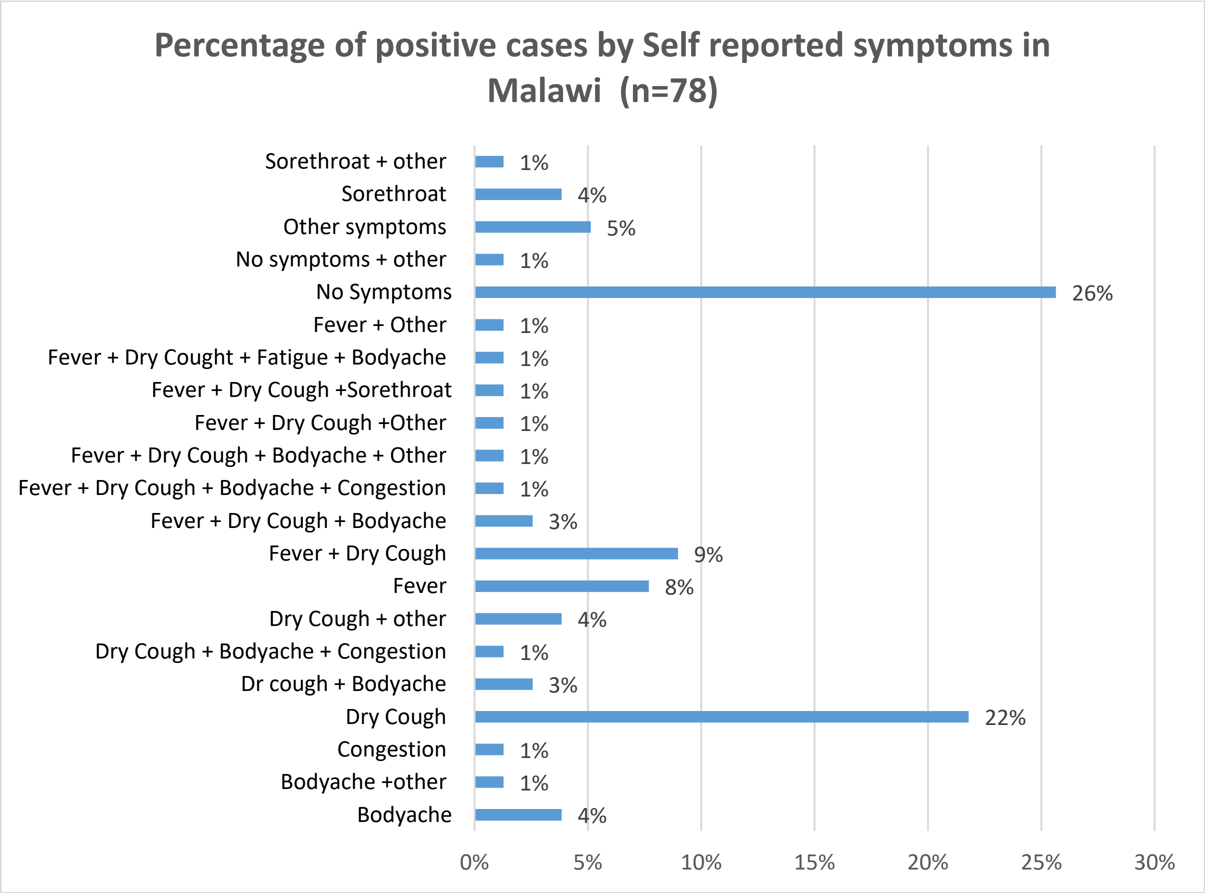

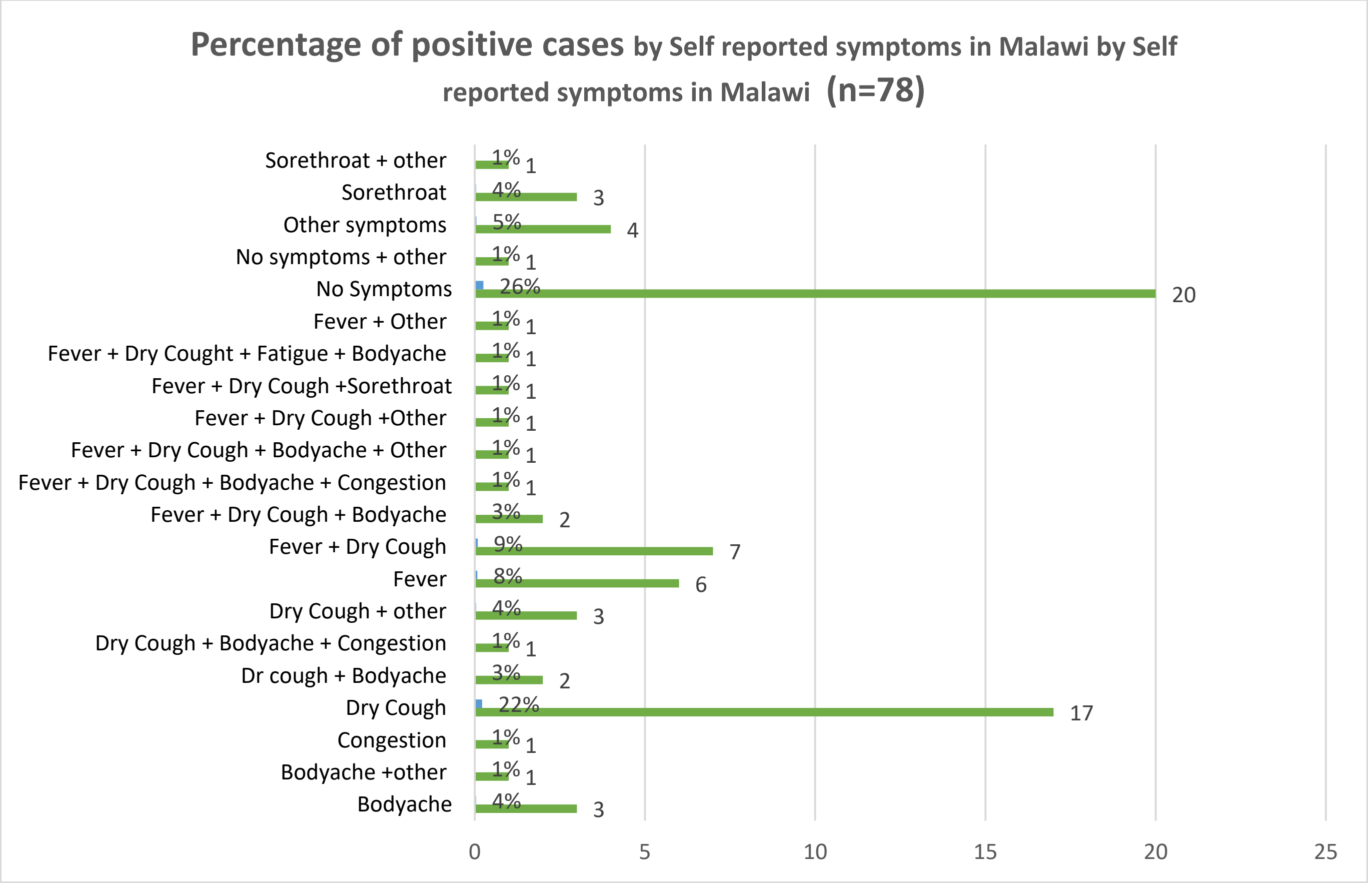

Supplementary Figure 2: SELF-Reported Symptoms of Individuals Who Tested at the Market in Zambia

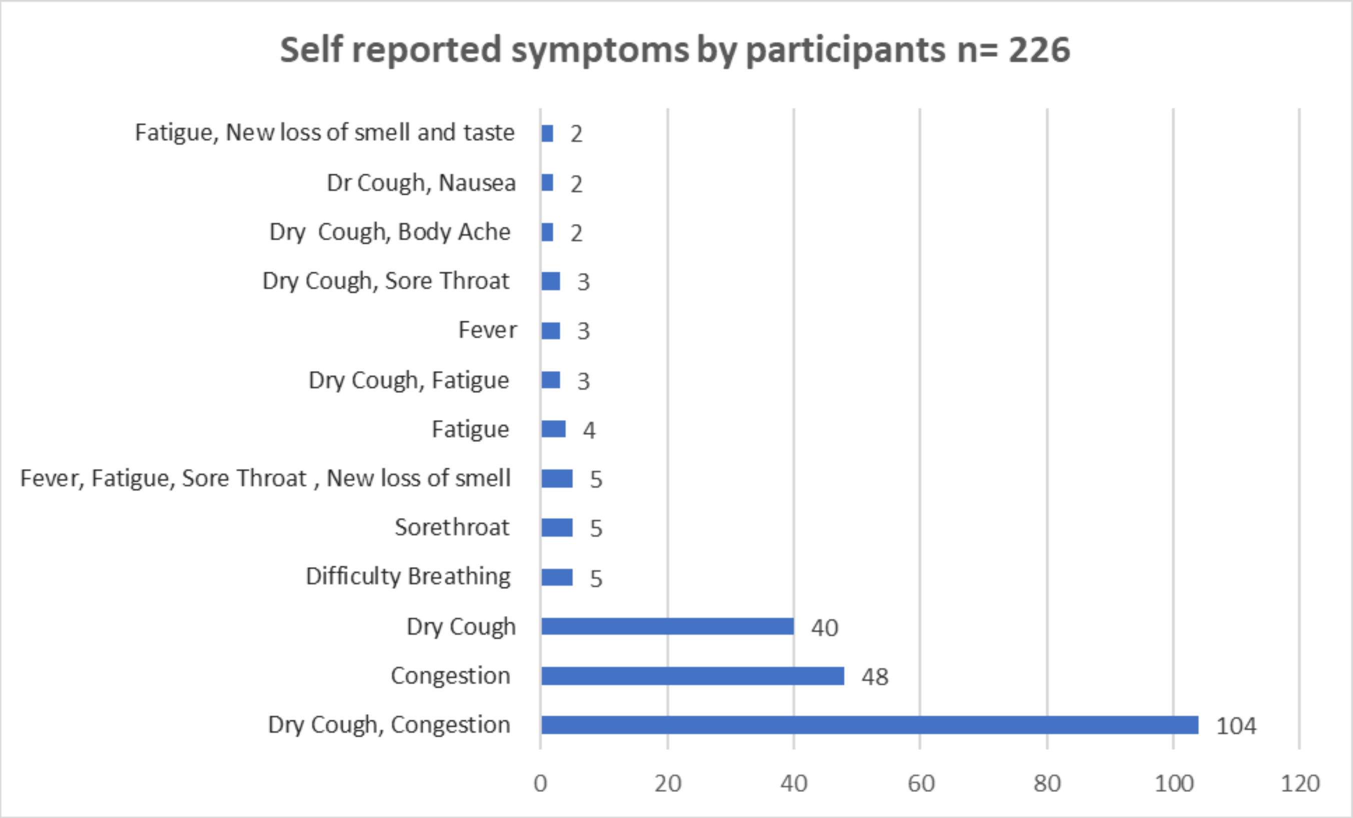

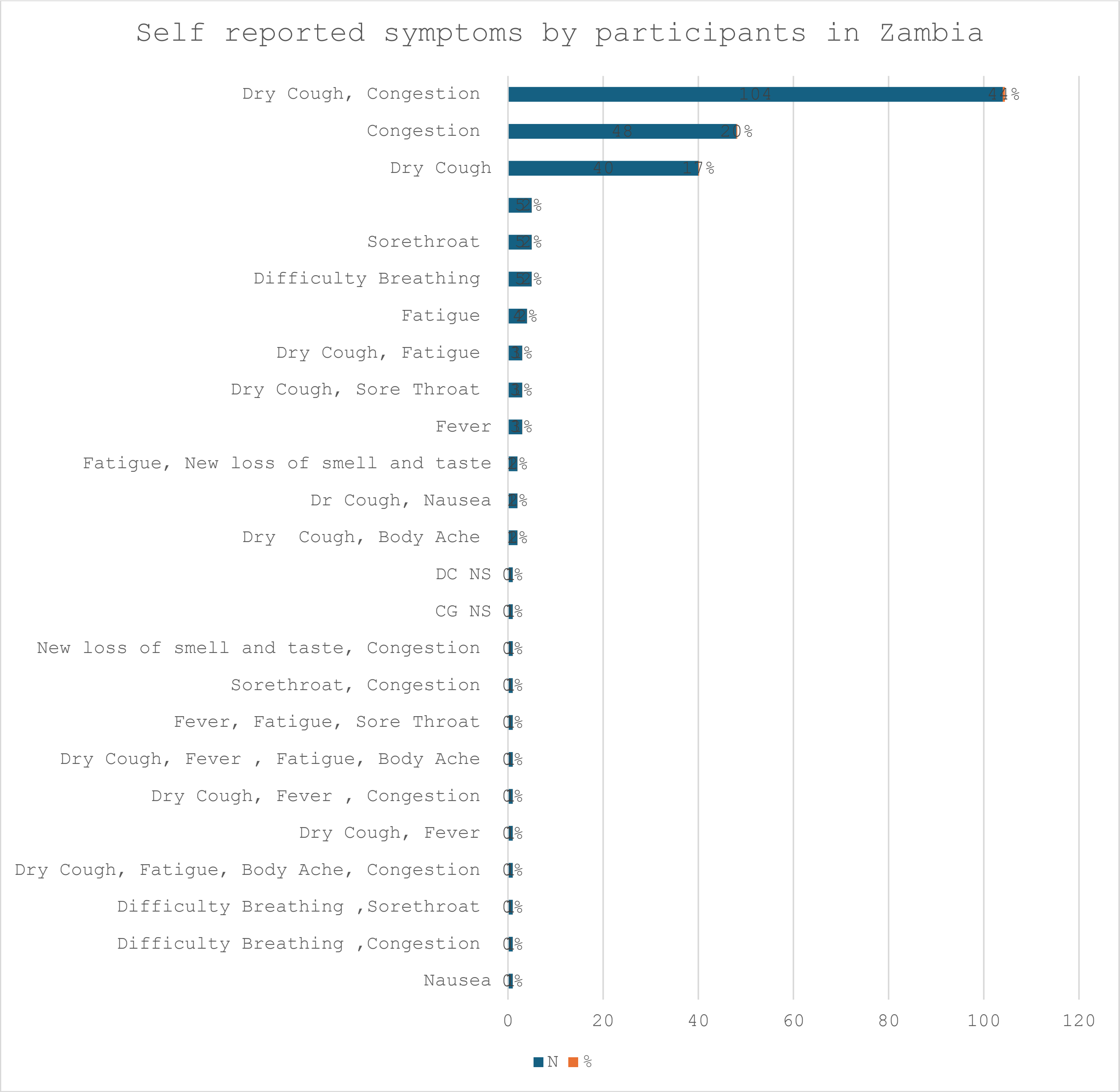
Self reported symptoms by participants in Zambia
Dry Cough, Congestion
Congestion
Dry Cough
Sorethroat
Difficulty Breathing
Fatigue
Dry Cough, Fatigue
Dry Cough, Sore Throat
Fever
Fatigue, New loss of smell and taste
Dr Cough, Nausea
Dry Cough, Body Ache
DC NS
CG NS
New loss of smell and taste, Congestion
Sorethroat, Congestion
Fever, Fatigue, Sore Throat
Dry Cough, Fever , Fatigue, Body Ache
Dry Cough, Fever , Congestion
Dry Cough, Fever
Dry Cough, Fatigue, Body Ache, Congestion
Difficulty Breathing ,Sorethroat
Difficulty Breathing ,Congestion
Nausea
104
44%
48
20%
40
17%
0
20
40
60
80
100
120
N
%

Malawi Questionnaires

1. Information Sheet and consent form for Healthcare Worker Questionnaires

Title of the proposed study: An Evaluation of Community-based Delivery and Administration of SARS-CoV-2 Antigen Rapid Tests

Investigators:

Background Purpose of the study:
This project led by the Ministry of Health (MOH) and Clinton Health Access Initiative (CHAI) aims to gather information on the implementation of a program involving point-of-care (POC) antigen testing for SARS-CoV-2, the virus causing the disease COVID-19. You are being invited to participate in a questionnaire because we are interested in the opinions and experiences of staff involved in POC antigen testing at this site.

Procedures:
You will be asked about your experience with the implementation of the POC COVID testing program at this site, the benefits, and challenges to the program, and the suggestions you have for improvements. The questionnaire will be read to you by a data collector and will last approximately 30-60 minutes during your working period

Risks/Discomforts:
Your position and your relationship with the MOH and CHAI will not be affected by whether or not you decide to participate in this questionnaire or by your responses. Your name and other personal information will not be requested and your participation in this study will be confidential.

Benefits:
Participation in this study may help to improve service delivery for people who wish to be tested for SARS CoV-2.

Compensation:
You will not be compensated for this questionnaire.

Questions:
Any individual who has complaints or questions about this study should contact:
Local PI name and contact information

Questions about participant rights:
For questions regarding your rights as a research participant contact:
Local PI name and contact information

Statement of voluntariness:
Participation in this questionnaire is completely voluntary. You may stop the questionnaire at any time to ask questions about participation or stop your participation completely. You can withdraw from the questionnaire at any point with no impact on your employment or performance. In the event that you withdraw your data and responses will be discarded and not kept on record.

Confidentiality:
Care will be taken to provide a private place to complete the questionnaire so that you feel free to speak. Questionnaire responses will be accessible only to the researchers and will not contain identifiable information. Specific information about you will never appear in a report or be published. Data collected will be stored on a secure, password-protected server and computer and only available to authorized CHAI staff. Your answers will be retained for three years after the end of the research project, or for a longer period if required by the local policy. Data from this study will be owned by each country's government and will be solely used to answer research questions around COVID-related policy.

STATEMENT OF CONSENT
I have been invited to participate in an evaluation of the operational assessment of the SARS-CoV-2 Antigen testing program. I have been selected to participate because I am a healthcare worker at this testing site. Participation will consist of a questionnaire that will last approximately 30-60 minutes. I understand that participation in this questionnaire is completely voluntary, and no compensation will be provided.

I have read the information about this study, or it has been read to me. I have had the opportunity to ask questions about it and any questions I have asked have been answered to my satisfaction. For any further questions, I may contact Andrews Gunda on +265888242894 and Joseph Bilitinyu-Bangor on +265999875399. I voluntarily consent to be a participant in this evaluation, and I understand that I can stop at any time or choose not to answer any questions.

Name…………………………………….
Signature of participant ……………………………………………….
Date (DD/MM/YY) ……………………………………………

Name………………………………………………..
Signature of Interviewer ………………………………………………….
Date (DD/MM/YY) ……………………………………………

2. Healthcare Worker questionnaire

Date: ___________________________________
Site: ________________________________
Gender: _________________________________
Age: ____________________________________

<u>Instructions:</u> The format of this data collection is a semi-structured questionnaire to be read aloud to the respondent and responses entered electronically onto Android tablets. Everyone will be invited and at minimum, **one questionnaire should be conducted with the person responsible for providing COVID-19 Antigen testing at the site** (or the acting in-charge, if the in-charge is not available). Administration of the questionnaire should take place in a private room so that staff feel comfortable answering freely and honestly. Questionnaires are meant to last approximately 30-60 minutes. Questionnaires refer to the COVID-19 Antigen testing.

1. What is your role at the testing site?
    - In-charge of site
    - Nurse
    - Lab technician
    - Community healthcare worker
    - Other, specify:

2. Have you received Training on COVID-19 antigen testing?
    - Yes
    - No

3. Please indicate how were you trained?
    - Official training
    - Unofficially trained by a peer
    - Other (Explain): _______________

4. Do you conduct COVID-19 testing at the site?
    - Yes
    - No

5. Have there been any patients who were eligible and willing to receive COVID-19 Antigen tests who did not receive them?
    - No
    - Yes. Specify why the patient did not receive the test:
        - Too many patients so unable to provide care
        - Not enough test kits

- Not enough staff
- Other

6. In the last week or 2 weeks, how long did **most** patients have to wait for their COVID-19 Antigen test results?
   - Less than 1 hour
   - 1-2 hours
   - 2-4 hours
   - 4-8 hours
   - 8-24 hours
   - 24 hours-1 week
   - More than 1 week

7. In the last week, did you have patients that have to wait for longer than 2 hours for their COVID-19 Antigen test results?
   - N0 (Skip to Q11)
   - Yes

8. In the last week, how often did the patients wait longer than 2 hours for their COVID-19 antigen test results?
   - Less than 1 out of every 10 patients
   - 1 to 3 out of every 10 patients
   - 4-6 out of every 10 patients
   - 7-10 out of every 10 patients
   - Others, specify
9. Have any patients expressed concerns about whether they can trust the results of the Antigen test because the results are available so rapidly?
   - No (Skip to Q13)
   - Yes, a few
   - Yes, many

10. Can you tell me more about the concerns of those patients? Note: Describe the comments made by the patients)
    - [__________]

11. Have site staff expressed concerns about whether they can trust the results?
    - None (Skip to Q15)
    - A few
    - Many

12. Can you tell me more about the concerns of those staff members?
    - [__________]

Prompt: Now I would like to ask you questions pertaining to how often the following issues in last week resulted in delays in returning test results?

13. High volume of COVID-19 Antigen tests requested at the site
    - Never
    - 1 time
    - 2-4 times
    - 4+ times

14. Limited number of trained operators to collect samples
    - Never
    - 1 time
    - 2-4 times
    - 4+ times

15. Limited number of trained testers to run the tests
    - Never
    - 1 time
    - 2-4 times
    - 4+ times

16. Stockouts of COVID-19 Antigen test kits
    - Never (Skip to Q21)
    - 1 time
    - 2-4 times
    - 4+ times

17. What was the average duration of the stockout?
    - Less than 1 day
    - 1 day to less than 1 week
    - 1 week to less than 1 month
    - 1 month or greater

18. How was the stockout fixed?
    - Waited until next scheduled shipment arrived
    - Partner provided extra shipment to fill the gap in stock
    - Other, specify:

19. Stockouts of PPE and other safety materials
    - Never (Skip to Q24)
    - 1 time
    - 2-4 times

- 4+ times

20. What was the average duration of the stockout?
    - Less than 1 day
    - 1 day to less than 1 week
    - 1 week to less than 1 month
    - 1 month or greater

21. How was the stockout fixed?
    - Waited until next scheduled shipment arrived
    - Partner provided extra shipment to fill the gap in stock
    - Other, specify:

22. Other reasons for COVID-19 Antigen test processing delays: (specify)
    - Never
    - 1 time
    - 2-4 times
    - 4+ times

23. What if any, factors are limiting the success of Rapid Antigen testing at this site?
    - [__________]

24. What, in your experience has been going well with regards to Rapid Antigen testing at this site?
    - [__________]

25. Do you have any ideas on how to make the Antigen testing program stronger at this site?
    - [__________]

3. Information Sheet and consent form for individual questionnaires

Title of the proposed study: An Evaluation of Community-based Delivery and Administration of SARS-CoV-2 Antigen Rapid Tests

Instructions for study staff: Please ask the participant to read this form carefully in either English or the local language based on their preference. If a participant cannot read or have trouble understanding, please read the information sheet to the participants in their preferred language.

Instructions for participants: Please read this form carefully or ask study staff to read the form to you. If you have any questions, please ask us. If you decide to participate in this research, you will be asked to sign this form. A copy of the signed form will be provided to you for your record.

Background & Purpose of the study: You are being invited to participate in a research study led by the Ministry of Health (MOH) and Clinton Health Access Initiative (CHAI) that is looking at ways to improve the SARS-CoV-2 Antigen testing program. This testing informs if you are infected and if you and your family members and contacts would need to quarantine to limit the spread of the virus. Here we will be asking questions to understand your testing experience at the testing site.

How many people will take part? Questionnaires will be offered to 10-15 participants in each site who have received an Antigen test.

Procedures: You will be asked about your experience with receiving the antigen test at this site. The questionnaire will last approximately 5-10 minutes.

Potential Risks:
The risks associated with participating in the study are considered to be minimal. Your name and other personal information will not be requested and your participation in this study will be confidential. Participation in the survey will not in any way affect the availability of your test result.

Potential Benefits:
This study has the potential to benefit you and the broader community by providing information that can be used to improve Antigen COVID-19 testing service delivery.

Compensation:
You will not be compensated for this study.

Voluntary participation:
Participation in this study is completely voluntary.

You are free to withdraw from this study at any time and for any reason. Refusal from the study will not have any impact on your access to testing, care, and treatment.

Questions:
Any individual who has complaints or questions about this study should contact:

Contact details of Principal investigator at CHAI Malawi	Contact details of principal investigator at Ministry of Health and Population.
Andrews Gunda<br>Clinton Health Access Initiative, Malawi.<br>Private Bag 341, Capital City<br>Lilongwe, Malawi.<br>Tel: +265888242894<br>Email: agunda@clintonhealthaccess.org	Joseph Bilitinyu-Bangoh<br>Ministry of Health and Population<br>P.O Box 30377, Capital City.<br>Lilongwe 3.<br>Tel: +265999875399<br>Email: jbb8871@hotmail.com

Questions about participant rights:
For questions regarding your rights as a research participant contact:

Contact details of Principal investigator at CHAI Malawi	Contact details of principal investigator at Ministry of Health and Population.
Andrews Gunda<br>Clinton Health Access Initiative, Malawi.<br>Private Bag 341, Capital City<br>Lilongwe, Malawi.<br>Tel: +265888242894<br>Email: agunda@clintonhealthaccess.org	Joseph Bilitinyu-Bangoh<br>Ministry of Health and Population<br>P.O Box 30377, Capital City.<br>Lilongwe 3.<br>Tel: +265999875399<br>Email: jbb8871@hotmail.com

Confidentiality:
Care will be taken to provide a private place to complete the questionnaire so that you feel free to speak. Questionnaire responses will be accessible only to the researchers and will not contain identifiable information. Specific information about you will never appear in a report or be published. Data collected will be stored on a secure, password-protected server and computer and only available to authorized CHAI staff. Your answers will be retained for three years after the end of the research project, or for a longer period if required by the local policy. Data from this study will be owned by each country's government and will be solely used to answer research questions around COVID-related policy.

Statement of consent:
I have been invited to participate in a questionnaire to assess my experiences with receiving tests for COVID-19 with an antigen test. I have been selected to participate because I have received an antigen

test at this testing site. Participation will consist of a questionnaire that will last approximately 5-10 minutes. I understand that participation in this questionnaire is completely voluntary, and no compensation will be provided.

I have read the information about this study, or it has been read to me. I have had the opportunity to ask questions about it and any questions I have asked have been answered to my satisfaction. For any further questions, I may contact Andrews Gunda on +265888242894 and Joseph Bilitinyu-Bangor on +265999875399. I voluntarily consent to be a participant in this study, and I understand that I can stop at any time or choose not to answer any questions.

**Name of Participant:**	**Name of Staff:**
______________________________	_____________________________________
**Signature of Participant:**	**Signature of Staff:**
__________________________	________________________________
**Date (DD/MM/YY):**	**Date (DD/MM/YY):**
_______________________________	_______________________________

If illiterate
A literate witness must sign (if possible, this person should be selected by the participant and should have no connection to the research team). Participants who are illiterate should include their thumbprint as well.

I have witnessed the accurate reading of the consent form to the potential participant, and the individual has had the opportunity to ask questions. I confirm that the individual has given consent freely.

Print name of witness: _______________________ **AND** **Thumb print of participant**
Signature of witness: ________________________
Date (DD/MM/YY): __________________________

4. Information Sheet and consent form for individual questionnaires (Chichewa)

***Mutu wa kafukufuku*:** An Evaluation of Community-based Delivery and Administration of SARS-CoV-2 Antigen Rapid Tests

Malangizo kwa ofunsa: *Chonde funsani wotenga mbali mu kafukufuku kuti awerenge fomu iyi mosamala muchingerezi kapena mu Chichewa malingana ndi momwe iwo akondela. Ngati wotenga mbali sakwanitsa kuwerenga kapena kapena akuvutika kumvesetsa, chonde awerengeleni muchiyankhulo chomwe iwo angamve.*

***Malangizo kwa otenga mbali*:** *Chonde werengani fomu iyi mosamala kapena afunseni otsogolera zokambilana kuti akuwerengereni. Ngati muli ndi mafunso ena aliwonse, chonde tifunseni. Ngati musankha kutenga nawo mbali mukafukufuku uyu muzafunsidwa kuti musaine fomu. Fomu yomwe muzasaine izaperekedwa kwa inu ngati umboni.*

Cholinga cha kafukufuku: Mukufunsidwa kutenga nawo mbali mukafukufuku yemwe akutsogozedwa ndi a unduna wa zaumoyo pamodzi ndi bungwe la CHAI yemwe akuyang'ana za njira zopititsa patsogolo kayezedwe ka matenda a Corona virus. Kuyeza uku kumadziwitsa ngati inu muli ndi kachilombo koyambitsa matenda a corona, komanso ngati inu ndi achibale anu ndi omwe mumalumikizana nawo muyenera kukhala panokha kuti muchepetse kufalikira kwa kachilomboka. Apa tikhala tikufunsa mafunso kuti timvetsetse zomwe mwakumana nazo pamalo oyezera.

Ndi anthu angati omwe atenge nawo mbali? Mafunso adzafunsidwa kwa anthu osachepera khumi komaso osapitilira khumi ndi asanu omwe ayezedwa kudzera munjira ya Antigen pamalo aliwonse oyezera.

Njira:. Mudzafunsidwa za zomwe mwakumana nazo pomwe mumayezedwa kudzera ndira ya Antigen pamalo pano. Mafunsowa atenga pafupifupi phindi khumi.

Zowopsa Zomwe Zingachitike:

Zowopsa zomwe zingachitike chifukwa chotenga nawo mbali mukafukufukuyu zitha kukhala zochepa. Dzina lanu ndi zina zanu sizizafunsidwa ndipo kutenga nawo mbali kwanu mukafukufuku uyu kudzakhala kwa chinsisi. Kutenga mbali mukafukufukuyu sikungasokoneza kupezeka kwa zotsatila za kuyezetsa kwanu.

Ubwino wotenga nawo mbali:

Kafukufuku uyu ali ndi kuthekera kopindulitsa inu ndi anthu ena onse popereka zambiri zomwe zingagwiritsidwe tchito popititsa patsogolo tchito zoyezetsa matenda a corona.

Malipiro:
Dziwani kuti kutenga nawo mbali mukafukufukuyu ndi kwa ulere ndipo simudzalipilidwa.

Kutenga nawo mbali mwakufuna kwanu:
Kutenga nawo mbali mukafukufuku uyu ndikodzifunila nokha.

Muli omasuka kusiya kutenga nawo mbali mukafukufukuyu nthawi ina iliyonse pazifukwa zilizonse. Kukana kutenga nawo mbali mukafukufukuyu sikudzakhuza mwayi wanu woyezetsa, chisamaliro komanso chithandizo.

Mafunso:
Aliyense amene ali ndi madandaulo kapena mafunso okhudza kafukufukuyu atumize uthenga ku:

Zambiri zolumikizana ndi wofufuza wamkulu ku bungwe la CHAI Malawi	Zambiri zolumikizana ndi wofufuza wamkulu ku Unduna wa Zaumoyo ndi Chiwerengero cha Anthu.
Andrews Gunda<br>Clinton Health Access Initiative, Malawi.<br>Private Bag 341, Capital City<br>Lilongwe, Malawi.<br>Tel: +265888242894<br>Email: agunda@clintonhealthaccess.org	Joseph Bilitinyu-Bangoh<br>Ministry of Health and Population<br>P.O Box 30377, Capital City.<br>Lilongwe 3.<br>Tel: +265999875399<br>Email: jbb8871@hotmail.com

Mafunso okhudza ufulu wa omwe atenge nawo mbali:
Pamafunso okhudzana ndi ufulu wanu ngati wochita nawo kafukufuku lemberani

Zambiri zolumikizana ndi wofufuza wamkulu ku bungwe la CHAI Malawi	Zambiri zolumikizana ndi wofufuza wamkulu ku unduna wa zaumoyo ndi chiwerengero cha anthu.
Andrews Gunda<br>Clinton Health Access Initiative, Malawi.<br>Private Bag 341, Capital City<br>Lilongwe, Malawi.<br>Tel: +265888242894<br>Email: agunda@clintonhealthaccess.org	Joseph Bilitinyu-Bangoh<br>Ministry of Health and Population<br>P.O Box 30377, Capital City.<br>Lilongwe 3.<br>Tel: +265999875399<br>Email: jbb8871@hotmail.com

Chinsisi:

Tidzayesetsa kupeza malo a chinsisi kuti mudzathe kuyankha mafunso komanso kuyankhula momasuka. Mayankho amafunso adzakhala ofikilidwa ndi otsogolera kafukufuku okhaokha ndipo sazakhala ndi chodziwika komwe akuchokera. Zambiri za inu sizizaoneka mu zolembedwa zochokera kukafukufukuyu kapena kutsindikizidwa. Zonse zochokera kukafukufukuyu zizasungidwa mosamala pa malo otetezedwa achinsisi ofikilapo okhawo ogwira tchito ovomelezeka a bungwe la CHAI. Mayankho anu adzasungidwa kwa zaka zitatu pambuyo pa kutha kwa kafukufukuyu, kapena kwa nthawi yotalikirapo ngati ndikofunika kutero. Zambiri zochokera mu kafukufukuyu zikhala za boma la dziko la Malawi ndipo zizigwiritsidwa ntchito poyankha mafunso ofufuza okhudzana ndi mfundo zokhudzana ndi COVID.

Chidziwitso cha chilolezo:
Ndayitanidwa kuti nditenge nawo mbali kuzokambilana za zomwe nakumana nazo poyezetsa matenda a COVID-19 kudzera njira ya Antigen. Ndasakhidwa kuti nditenge nawo mbali chifukwa ndayezedwa kudzera njira ya Antigen pamalo ano oyezera. Kutenga nawo gawo kudzakhala ndi mafunso omwe atenga pafupifupi mphindi 5-10. Ndikumvesetsa kuti kutenga nawo mbali muzokambilanazi ndikosakakamiza ndipo sindizalipilidwa kalikonse.

Ndawerenga zambiri za kafukufukuyu, kapena zawerengedwa kwa ine. Ndakhala ndi mwayi wofunsa mafunso okhudza izi ndipo mafunso aliwonse omwe ndafunsa ayankhidwa mokhutiritsidwa. Pamafunso ena aliwonse, ndingalumikizane ndi Andrews Gunda pa +265888242894 ndi Joseph Bilitinyu -Bangor pa +265999875399. Ndikuvomera mwakufuna kwanga kutenga nawo mbali mu kafukufukuyu, ndipo ndikumvetsa kuti ndikhoza kuyima nthawi ina iliyonse kapena kusankha kusayankha mafunso aliwonse.

Dzina la otenga mbali: ___________________________

Siginecha ya otenga mbali: _______________________

Tsiku (DD/MM/YY): ____________________________

Dzina la otsogolera zokambilana: _________________________________

Siginecha ya otsogolera zokambila: _____________________________

Tsiku (DD/MM/YY): ____________________________

Ngati sadziwa kuwerenga
Mboni yodziwa kulemba ndi kuwerenga iyenera kusaina (Ngati mkotheka munthuyu asankhidwe ndi otenga nawo mbali ndipo asakhale ndi mgwirizano wina uliwonse ndi ofunsayo). Otenga nawo mbali amene sadiwa kulemba ndi kuwerenga ayeneranso kutsindikiza chala chawo chachikulu.

Ndachitira umboni kuwerenga molondola kwa fomu yachilolezo kwa omwe akutenga nawo mbali, ndipo munthuyu wakhala ndi mwayi wofunsa mafunso. Ndikutsimikizira kuti munthuyo wapereka chilolezo mosakakamizidwa

Dzina la ochitira umboni: ______________________ **AND** **Thumb print of participant**

Siginecha ya ochitira umboni: ______________________
Tsiku (DD/MM/YY): ________________________

5. Individual questionnaire

Date: ___________________________________
Testing site: _________________________________
Antigen test used: ________________________
Test Result: ______________________________
Gender: _________________________________
Age: ____________________________________
Symptoms: ______________________________

Instructions: The format of this data collection is a structured questionnaire to be read aloud to the respondent and responses entered electronically onto Android tablets. Questionnaire should be conducted with the individual receiving a COVID-19 Antigen testing. The questionnaire should take place in a private room so that individual feels comfortable answering freely and honestly. Questionnaires are meant to last approximately 5-10 minutes.

1. Before today, have you previously been tested for COVID-19?
   - Yes
   - No (Skip to Q6)

2. When did you receive your last test?
   - ______________(DD/MM/YYY)

3. Where did you get tested for COVID-19?
   - At the quarantine zone
   - At the hospital
   - At a MoH COVID -19 testing site
   - At border post
   - At the airport
   - At a community testing site
   - Other, specify ______

4. What was the result?
   - Positive
   - Negative
   - Invalid/Indeterminate
   - I don’t know/Prefer not to say

5. Which testing method was used?
   - Don’t know/Prefer not to say
   - RT-PCR

- Antibody test
- Antigen test
- Other, specify

6. How did you hear about COVID-19 testing was being offered at this site?
   - From HCW at the site
   - From local healthcare facility or hospital
   - From the announcement (newspaper, radio)
   - From a poster at the entrance to the site
   - From a friend
   - Other, specify
   - No response

7. Why did you choose to participate in testing today?
   - Had some symptoms
   - Curiosity
   - Have recently been in contact with someone who tested positive
   - To take good care of my health
   - Other, specify ________________________

8. Did you find the sample collection experience acceptable?
   - Very acceptable
   - Acceptable
   - Neutral
   - Not acceptable Specify reason: ________________________

9. Did you find the overall testing experience acceptable?
   - Very acceptable
   - Acceptable
   - Neutral
   - Not acceptable Specify reason: ________________________

10. After your sample was taken today, how long did it take to receive your COVID-19 test result?
    - Same day: less than 1 hours
    - Same day: 1 -2 hours
    - Same day: more than 2 hours
    - Was told to come back the next day
    - Never received

11. What's the maximum length of time that you desire for a COVID-19 test result to be received?
    - Same day: less than 1 hours
    - Same day: 1 to 2 hours

- Same day: more than 2 hours
- Next Day
- Within 1 week
- At the next appointment (14 days)
- Other, specify____________

12. Do you trust the COVID-19 test result?
    - Yes
    - No, Why not?: ______________________________

13. If you receive a positive test result today, how likely is it that you will quarantine for the recommended 14 days? Please feel free to specify your reason
    - Highly unlikely
    - Unlikely
    - Neither likely not unlikely
    - Likely
    - Highly likely
        - Reason for answer ______________________________
    - Decline to answer

14. Would you consider this COVID test in the future when you need to be screened for COVID-19?
    - Yes
    - No, specify: _______________________________

15. What do you think worked well about the COVID services you received today?
    - Open ended: ___________________________________

16. Overall, would you recommend COVID testing at this testing site to your family and friends?
    - Yes
    - No, specify: ____________________________________

17. Do you have any suggestions on how we can improve the testing service?
    - Open ended: __________________________________

6. Individual questionnaire (Chichewa)

Tsiku: ________________________________
Malo oyezera: ______________________________
Ntundu wa Antigen test womwe wagwiritsidwa tchito: ______________________
Zotsatira: ___________________________
Mayi/Bambo: ______________________________
Zaka: _________________________________
Zizindikiro: ____________________________

Malangizo: Mafunso azawerengedwa kwa otenga mbali ndi mayankho onse azalowetsedwa pa zipangizo za Android. Mafunso ayenera kufunsidwa kwa amene ayezedwa kugwiritsa tchito njira ya Antigen. Zokambilana ziyenera kuchitikira m'chipinda chachinsisi kuti munthu atha kuyankha mafunso momasuka komanso moona mtima. Zokambilana ziyenera kukhala pafupifupi mphindi 5-10.

Kupatulapo lero, kodi m'bumyomu munayezetsapo COVID-19?

- Inde
- Ayi (Pitani ku funso Q6)

1. Kodi ndi liti lomwe munayezetsa matenda a COVID-19 mmbuyomu?
   - ____________(DD/MM/YYY)

2. Munayezetsa kuti COVID-19?
   - Ku quarantine zone
   - Kuchipatala
   - Pamalo oyezera COVID-19 okhazikitsidwa ndi a Unduna wa Zaumoyo
   - Pamalo otulukira komanso olowera mdziko.
   - Pabwalo la ndege
   - Pamalo oyezetsa anthu a mmudzi
   - Ena, Tchulani _____

3. Kodi zotsatira zake zinali zotani?
   - Anandipeza ndi COVID-19
   - Sanandipeze ndi COVID-19
   - Zosavomerezeka/Zosatsimikizika
   - Sindikudziwa/Sindingathe kunena

4. Kodi ndi njira iti yoyesera yomwe idagwiritsidwa tchito?
   - Sindikudziwa/Sindingathe kunena
   - RT-PCR
   - Kuyesa kwa Antibody
   - Kuyesa kwa Antigen
   - Zina, Tchulani

5. Kodi munadziwa bwanji kuti kuyeza matenda a COVID-19 kukuchitika pamalo pano ?
   - Kuchokera kwa ogwira tchito wa zaumoyo pamalo pano.
   - Kuchokera kuchipatala chamdera lino
   - Kuchokera kuzolengeza (Nyuzipepala, wailesi)
   - Kuchokera pazithunzi zoikidwa malo oyezetsera.
   - Kuchokera kwa nzanga
   - Malo ena, Tchulani
   - Palibe yankho

6. Mchifukwa chiyani mwasankha kutenga nawo mbali pakuyezetsa lero?
   - Ndinali ndi zizindikiro.
   - Ndinali ndi chidwi
   - Posachedwa ndakumana ndi munthu yemwe adapezeka ndi kachilombo ka COVID-19.
   - Kusamalila Thanzi langa
   - Zifukwa zina, Tchulani ________________________

7. Kodi mwapeza kutenga zoti akayeze kovomerezeka?
   - Zovomerezeka Kwambiri
   - Zovomerezeka
   - Pakatikati
   - Zosavomelezeka, Nenani zifukwa: ________________________

8. Kodi mwapeza kuyezetsa konse kuti ndikovomerezeka?
   - Zovomerezeka kwambiri
   - Zovomerezeka
   - Pakatikati
   - Zosavomerezeka Nenani zifukwa: ________________________

9. Kuchokera pomwe anakuyezani lero, zatenga nthawi yaitali bwanji kuti mulandile zotsatira zanu?
   - Tsiku lomwelo: osakwana ola limodzi
   - Tsiku lomwelo: Osachepera ola limodzi komanso osapitilira maola awiri
   - Tsiku lomwelo: Kupitilila maola awiri
   - Ndinauzidwa kuti ndibwerenso tsiku losatira
   - Sindinalandile zotsatira konse

10. Kodi ndi nthawi yotalika bwanji yomwe mungakonde kuti muzilandila zotsatira za kuyeza matenda a COVID-19?
   - Tsiku lomwelo: osakwana ola limodzi
   - Tsiku lomwelo: Osachepera ola limodzi komanso osapitilira maola awiri
   - Tsiku lomwelo: kupitilira maola awiri
   - Tsiku losatira

- Pasanathe sabata limodzi
- Panthawi yotsatira (matsiku 14)
- Zina, tchulani___________

11. Kodi mumakhulupilira zotsatira za kuyeza COVID-19?
    - Inde
    - Ayi, Chifukwa chiyani?: ____________________________

12. Mutapezeka ndi matenda a COVID-19 lero, pali mwayi wochuluka bwanji kuti mungakhale kwa nokha kwa masiku khumi ndi anayi ovomerezeka? Chonde khalani womasuka kufotokoza zifukwa zake.
    - Zokayikitsa kwambiri
    - Zokayikitsa
    - Mwina zingachitike kapena ayi
    - Zingachitike
    - Zotheka kwambiri
        - Chifukwa cha yankho ____________________________
    - Sindingathe kuyankha

13. Kodi mtsogolomu mukadzafuna kuyezetsa matenda a COVID-19, mungafune kuyezedwa COVID-19 kugwiritsa tchito njira yomwe agwiritsa tchito azaumoyo lero?
    - Inde
    - Ayi, nenani zifukwa: _____________________________

14. Kodi mukuganiza kuti zinayenda bwino ndi chani pakuyezedwa COVID-19 mwalandila lero?
    - Atha kuyankhula zakukhosi mopanda malire: _________________________________

15. Kodi mungalimbikitse anzanu kapena apabanja panu kubwera kuzayezetsa matenda a COVID-19 pamalo pano?
    - Inde
    - Ayi , nenani zifukwa: __________________________________

16. Kodi mungakhale ndi malingaliro omwe mukuona ngati tingasinthire kapena kupititsa patsogolo tchito zoyesa?
    - Atha kuyankha zakukhosi mopanda malire: ________________________________

7. Information Sheet and consent form for post-test follow-up call for individuals who test positive

Title of the proposed study: Evaluation of Community-based Delivery and Administration of SARS-CoV-2 Antigen Rapid Tests in Malawi and Zambia

Instructions for study staff: Please ask the participant to read this form carefully in either English or the local language based on their preference. If a participant cannot read or have trouble understanding, please read the information sheet to the participants in their preferred language.

Instructions for participants: Please read this form carefully or ask study staff to read the form to you. If you have any questions, please ask us. If you decide to participate in this part of the research, you will be asked to sign this form and provide a phone number that we can contact you on. A copy of the signed form will be provided to you for your record.

Background & Purpose of the study: You are being invited to participate in a research study led by the Ministry of Health (MOH) and Clinton Health Access Initiative (CHAI) that is looking at ways to improve the SARS-CoV-2 Antigen testing program. This testing informs if you have SARS-CoV-2 and success of self-quarantine after testing positive. Here we will be asking questions to understand your experience with quarantine after receiving a positive antigen result at the end of the required 14-day period.

Who will take part? Follow-up calls on day 14 after receiving the positive result will be offered to **all participants in each site who have received a positive antigen test.**

Procedures: You will be asked over the phone about your experience with quarantine after receiving the antigen test at this site. The phone call will last approximately 5-10 minutes.

Potential Risks:
The risks associated with participating in the study are minimal. Your name will not be requested and your participation in this study will be confidential.
However, we will need a phone number to be able to reach you after the designated quarantine period.
Participation in the survey will not in any way affect any future access to testing or medical treatment.

Potential Benefits:
This study has the potential to benefit you and the broader community by providing information that can be used to improve antigen COVID-19 testing service delivery and COVID-19 surveillance and prevention measures.

Compensation:
You will not be compensated for this follow-up call.

Voluntary participation:
Participation in this Post-test survey follow-up call is voluntary. You are free to withdraw from the call at any time and for any reason.

Refusal to receive the post-test follow up call will not have any impact on your access to testing, care, and treatment in the future

Questions:
Any individual who has complaints or questions about this study should contact:

Contact details of Principal investigator at CHAI Malawi	Contact details of principal investigator at Ministry of Health and Population.
Andrews Gunda<br>Clinton Health Access Initiative, Malawi.<br>Private Bag 341, Capital City<br>Lilongwe, Malawi.<br>Tel: +265888242894<br>Email: agunda@clintonhealthaccess.org	Joseph Bilitinyu-Bangoh<br>Ministry of Health and Population<br>P.O Box 30377, Capital City.<br>Lilongwe 3.<br>Tel: +265999875399<br>Email: jbb8871@hotmail.com

Questions about participant rights:
For questions regarding your rights as a research participant contact:

Contact details of Principal investigator at CHAI Malawi	Contact details of principal investigator at Ministry of Health and Population.
Andrews Gunda<br>Clinton Health Access Initiative, Malawi.<br>Private Bag 341, Capital City<br>Lilongwe, Malawi.<br>Tel: +265888242894<br>Email: agunda@clintonhealthaccess.org	Joseph Bilitinyu-Bangoh<br>Ministry of Health and Population<br>P.O Box 30377, Capital City.<br>Lilongwe 3.<br>Tel: +265999875399<br>Email: jbb8871@hotmail.com

Confidentiality:
Care will be taken to provide a private place to complete the questionnaire so that you feel free to speak. Questionnaire responses will be accessible only to the researchers and will not contain identifiable information. Specific information about you will never appear in a report or be published. Data collected will be stored on a secure, password-protected server and computer and only available to authorized CHAI staff. Your answers will be retained for three years after the end of the research project, or for a longer period if required by the local policy. Data from this study will be owned by each country's government and will be solely used to answer research questions around COVID-related policy.

Statement of consent:

I have been invited to participate in an evaluation of the operational assessment of the SARS-CoV-2 Antigen testing study. I have been selected to participate because I received an antigen test at this testing site. Participation will consist of a follow-up post-test survey call after the designated 14 days of quarantine. The phone call will last approximately 5-10 minutes. I understand that participation in this questionnaire is completely voluntary, and no compensation will be provided.

I have read the information about this study, or it has been read to me. I have had the opportunity to ask questions about it and any questions I have asked have been answered to my satisfaction. For any further questions, I may contact the study investigators. I voluntarily consent to be a participant in this evaluation, and I understand that I can stop at any time or choose not to answer any questions. For any further questions, I may contact Andrews Gunda on +265888242894 and Joseph Bilitinyu-Bangor on +265999875399. I voluntarily consent to be a participant in this study, and I understand that I can stop at any time or choose not to answer any questions.

Name of Participant: ________________________

Name of Staff: ______________________________

Signature of Participant: _____________________

Signature of Staff: ___________________________

Date (DD/MM/YY): _________________________

Date (DD/MM/YY): _________________________

Phone number to contact: ____

Indicate best time for a follow-up call: _________________________________

If illiterate

A literate witness must sign (if possible, this person should be selected by the participant and should have no connection to the research team). Participants who are illiterate should include their thumbprint as well.

I have witnessed the accurate reading of the consent form to the potential participant, and the individual has had the opportunity to ask questions. I confirm that the individual has given consent freely.

Print name of witness: _____________________ **AND** **Thumb print of participant**

Signature of witness: _____________________

Date (DD/MM/YY): ________________________

8. Information Sheet and consent form for post-test follow-up call for individuals who test positive (Chichewa)

Mutu wa kafukufuku: Evaluation of Community-based Delivery and Administration of SARS-CoV-2 Antigen Rapid Tests in Malawi and Zambia.

Malangizo kwa ofunsa :. Chonde funsani otenga mbali kuti awerenge fomuyi mosamala muchingerezi kapena Chichewa kutengera ndi kukonda kwao. Ngati otenga mbali sangakwanitse kuwerenga kapena akuvutika kumvesetsa, chonde werengani fomuyi kwa otenga mbali muchiyankhulo chomwe iwo angakonde.

Malangizo kwa otenga mbali: Chonde werengani fomuyi mosamala kapena funsani wotsogolera zokambilana kuti akuwerengereni fomuyi. Ngati muli ndi mafunso, chonde tifunseni. Ngati mwaganiza kutenga nawo mbali mu gawo ili la kafukufukuyu, mudzafunsidwa kusaina fomu ndikupereka nambala yafoni yomwe tingakulumikizani nayo. Fomu yosainidwa lidzaperekedwa kwa inu ngati umboni.

Cholinga cha kafukufuku: Mukuitanidwa kutenga nawo mbali mu kafukufuku wotsogozedwa ndi Unduna wa Zaumoyo (MOH) ndi bungwe la Clinton Health Access Initiative (CHAI) yemwe akuyang'ana njira zowongolera pulogalamu yoyezetsa matenda a COVID-19 ya Antigen . Kuyeza uku kumadziwitsa ngati muli ndi SARS-CoV-2 komanso podzipatula mutapezeka ndi matenda a COVID-19. Tikhala tikukufunsani mafunso kuti timvetsetse zomwe mwakumana nazo pozipatula mutapezeka ndi matenda a COVID-19 kumapeto kwa nthawi yofunikira ya masiku 14.

Ndani atenge nawo mbali? Onse amene apezeka ndi matenda a COVID-19 azatsatiridwa patatha masiku 14 kudzera kunjira ya lamya.

Njira: Muzafunsidwa kudzera pa lamya za zomwe mwakumana nazo pozipatula mutapezeka ndi matenda a COVID-19 kuchoka pamalo oyezera ano. Kucheza kwa pa lamya kuzatenga phindi pakatikati zisanu kufikira khumi.

Zowopsa zomwe zingachitike: Zowopsa zomwe zingachitike chifukwa chotenga nawo mbali mu kafukufukuyu ndizochepa. Dziwani kuti dzina lanu silizafunsidwa ndipo kutenga mbali mukafukufukuyu kudzakhala kwa chinsisi. Koma dziwani kuti tifuna nambala yanu ya foni kuti tizathe kukufikilani pakatha nthawi yoikidwiratu yoti mukhale panokha. Kutenga nawo mbali mukafukufukuyu sikungakhudze mwayi wina uliwonse wamtsgolo wokayezetsa kapena kulandila chithandizo chamakhwala.
Ubwino ungakhalepo:
Kafukufuku uyu ali ndi kuthekera kopindulira inu komaso anthu ena onse popereka zambiri zomwe zingagwiritsidwe tchito popititsa patsogolo tchito zoyezetsa komaso kupewa COVID-19.

Malipiro:
Dziwani kuti kutenga nawo mbali mukafukufuku uyu ndikwaulere ndipo simuzapatsidwa malipiro ena aliwonse.

Kutenga nawo mbali mwakufuna kwanu:
Kutenga nawo mbali mukafukufuku uyu ndikosakakamiza. Ndinu omasuka kusiya kuyankha foni panthawi ina iliyonse komaso pa zifukwa zilizonse.

Kukana kulandira foni yotsatira pambuyo pakayezedwe ka COVID-19 sikungakhudze mwayi wanu woyezetsa, chisamaliro, ndi chithandizo mtsogolo.

Mafunso:
Aliyense amene ali ndi madandaulo kapena mafunso okhudza kafukufukuyu atumize uthenga ku:

Zambiri zolumikizana ndi wofufuza wamkulu ku bungwe la CHAI Malawi.	Zambiri zolumikizana ndi wofufuza wamkulu ku Unduna wa Zaumoyo ndi Chiwerengero cha Anthu.
Andrews Gunda<br>Clinton Health Access Initiative, Malawi.<br>Private Bag 341, Capital City<br>Lilongwe, Malawi.<br>Tel: +265888242894<br>Email: agunda@clintonhealthaccess.org	Joseph Bilitinyu-Bangoh<br>Ministry of Health and Population<br>P.O Box 30377, Capital City.<br>Lilongwe 3.<br>Tel: +265999875399<br>Email: jbb8871@hotmail.com

Mafunso okhudza ufulu wa omwe atenge nawo mbali:
Pamafunso okhudzana ndi ufulu wanu ngati wochita nawo kafukufuku lemberani:

Zambiri zolumikizana ndi wofufuza wamkulu ku bungwe la CHAI Malawi	Zambiri zolumikizana ndi wofufuza wamkulu ku Unduna wa Zaumoyo ndi Chiwerengero cha Anthu.
Andrews Gunda<br>Clinton Health Access Initiative, Malawi.<br>Private Bag 341, Capital City<br>Lilongwe, Malawi.<br>Tel: +265888242894<br>Email: agunda@clintonhealthaccess.org	Joseph Bilitinyu-Bangoh<br>Ministry of Health and Population<br>P.O Box 30377, Capital City.<br>Lilongwe 3.<br>Tel: +265999875399<br>Email: jbb8871@hotmail.com

Chinsisi:
Tidzayesetsa kupeza malo a chinsisi kuti mudzathe kuyankha mafunso komanso kuyankhula momasuka. Mayankho amafunso adzakhala ofikilidwa ndi otsogolera kafukufuku okhaokha ndipo sazakhala ndi

chodziwika komwe akuchokera. Zambiri za inu sizizaoneka mu zolembedwa zochokera kukafukufukuyu kapena kutsindikizidwa. Zonse zochokera kukafukufukuyu zizasungidwa mosamala pa malo otetezedwa achinsisi ofikilapo okhawo ogwira tchito ovomelezeka a bungwe la CHAI. Mayankho anu adzasungidwa kwa zaka zitatu pambuyo pa kutha kwa kafukufukuyu, kapena kwa nthawi yotalikirapo ngati ndikofunika kutero. Zambiri zochokera mu kafukufukuyu zikhala za boma la dziko la Malawi ndipo zizigwiritsidwa ntchito poyankha mafunso ofufuza okhudzana ndi mfundo zokhudzana ndi COVID.

Chidziwiso cha chililezo:

Ndaitanidwa kuti nditenge nawo mbali pa kafukufuku wokhuzana ndi matenda a COVID-19. Ndasankhidwa kuti nditenge nawo mbali chifukwa ndinayesedwa matenda a COVID-19 pamalo pano. Kutenga mbali kudzakhala ndi kundiyimbila foni patatha masiku 14 ovomerezeka kuti ndikhale pandekha atandipeza ndi matenda a COVID-19. Kucheza kodzera pa foni kudzatenga phindi pakati pa zisanu ndi khumi. Ndikumvesetsa kuti kutenga nawo mbali mukafukufukuyu ndikosakakamiza ndipo sipadzakhala malipilo ena aliwose.

Ndawerenga zambiri za kafukufukuyu, kapena zawerengedwa kwa ine. Ndakhala ndi mwayi wofunsa mafunso okhudza izi ndipo mafunso aliwonse omwe ndafunsa ayankhidwa mokhutiritsidwa. . Pamafunso ena aliwonse, nditha kulumikizana ndi otsogolera kafukufukuyu. Ndikuvomeleza mwandekha kuti ndikhale wotenga nawo mbali pa kafukufukuyu ndipo ndikumvesetsa kuti nditha kusiya kutenga nawo mbali panthawi inailiyonse komaso nditha kusankha kusayankha mafunso ena. Pamafunso ena aliwonse, ndingalumikizane ndi Andrews Gunda pa +265888242894 ndi Joseph Bilitinyu -Bangor pa +265999875399. Ndikuvomera mwakufuna kwanga kutenga nawo mbali mu kafukufukuyu, ndipo ndikumvetsa kuti ndikhoza kuyima nthawi ina iliyonse kapena kusankha kusayankha mafunso aliwonse.

Dzina la Otenga mbali: ________________________

Siginecha ya otenga mbali : _____________________

Tsiku (DD/MM/YY): _________________________

Dzina la otsogolera zokambilana: _____________________________

Siginecha ya otsogolera zokambilana: __________________________

Tsiku (DD/MM/YY): _________________________

Nambala ya foni ya otenga mbali: ____

Onetsani nthawi yabwino yoyimbira foni: ________________________________

Ngati sadziwa kuwerenga

Mboni yodziwa kulemba ndi kuwerenga iyenera kusaina (Ngati mkotheka munthuyu asankhidwe ndi otenga nawo mbali ndipo asakhale ndi mgwirizano wina uliwonse ndi ofunsayo). Otenga nawo mbali amene sadiwa kulemba ndi kuwerenga ayeneranso kutsindikiza chala chawo chachikulu.

Ndachitira umboni kuwerenga molondola kwa fomu yachilolezo kwa omwe akutenga nawo mbali, ndipo munthuyu wakhala ndi mwayi wofunsa mafunso. Ndikutsimikizira kuti munthuyo wapereka chilolezo mosakakamizidwa

Dzina la mboni: ______________________ AND Chidindo cha chala chachikulu
Siginecha ya mboni: _______________________
Tsiku (DD/MM/YY): _________________________

9. Post Test survey

Date of interview : ___________________________________
Setting site : _________________________________
Date of test: __________________________________
Antigen test used: ________________________
Test Result: ______________________________
Gender: _________________________________
Age: ____________________________________
Symptoms: _______________________________

<u>Instructions:</u> The call is a quick follow-up survey on the COVID-19 test you received at XXXX market. The information collected will be confidential and anonymous for operational research purposes.

1. Do you agree to speak with us about your quarantine experience?
   - Yes
   - No (Stop the interview)
     i. Would you like to give a reason for not completing the interview? _________

2. Do you remember which testing method was used?
   - Don’t know/Prefer not to say
   - RT-PCR
   - Antibody test
   - Antigen test
   - Other, specify

3. What was your test result?
   - Positive
   - Negative
   - Invalid/Indeterminate
   - I don’t know/Prefer not to say

4. How many individuals live in your household?
   - Specify: __________

5. Did anyone else in your household test positive for COVID?
   - Yes
   - No

6. How many days total did you stay isolated?
   - 1-3 days
   - 4-5 days
   - 6-10 days
   - 11-14 days
   - Refused to answer
   - Other

7. Did you take any other preventive actions after testing positive?
   - Wearing mask
   - Working from home
   - Informed Contacts
   - Other, specify _________

8. Did anyone else in household test positive for COVID?
   - Yes
   - No

9. Did you find quarantine at home to be a challenge?
   - Yes
   - No

10. If yes to the previous question can you, please describe the challenges you faced __________

11. Do you have any suggestions on how we can improve quarantine to limit transmission of COVID-19?
    - Open-ended

10. Post Test survey (Chichewa)

Tsiku : ____________________________________

Malo : __________________________________

Tsiku loyezetsa: ___________________________________

Ntundu wa Antigen amene wagwiritsidwa tchito : ________________________

Zotsatira zoyesa: ______________________________
Amayi/Abambo: _________________________________
Zaka: ____________________________________
Zizindikiro: _______________________________

Malangizo: Ndakuimbilani foni kuti ndipange kafukufuku pambuyo pa Kuyezetsa matenda a COVID-19 komwe kunachitika pa malo a XXXX. Zomwe titakambilane zizakhala zachinsinsi komaso zosadziwika pakafukufukuyu.

1. Kodi mukuvomereza kuyankhulana nafe zokhuzana ndi zomwe mwakumana nazo nthawi yomwe mwakhala panokha?
   - Inde
   - Ayi (Imitsani zokambilana)
     i. Kodi mungakonde kupereka chifukwa chosamaliza kuyankhulana? _________

2. Mukukumbukira njira yoyesera matenda a COVID-19 yomwe inagwiritsidwa tchito pokuyesani?
   - Sindikudziwa/Sindingathe kunena
   - RT-PCR
   - Njira ya Antibody
   - Njira ya Antigen
   - Njira zina, tchulani

3. Kodi zotsatira zanu zinali zotani?
   - Anandipeza ndi matenda a COVID-19
   - Sanandipeze ndi matenda a COVID-19
   - Zosavomerezeka/Zosatsimikizika
   - sindikudziwa/Sindingathe kunena

4. Ndi anthu angati omwe amakhala m'nyumba mwanu?
   - Tchulani nambala: _________

5. Kodi pali wina m'banja mwanu yemwe adapezeka ndi matenda a COVID-19?
   - Inde
   - Ayi

6. Kodi mwakhala nokha kwamasiku angati chifukwa chopezeka ndi matenda a COVID-19?
   - 1-3 days
   - 4-5 days
   - 6-10 days
   - 11-14 days
   - Anakana kuyankha

- Ena

7. Kodi munachitapo zodzitetezera mutapezeka ndi matenda a COVID-19?
    - Kuvala masiki
    - Kugwirira tchito kunyumba
    - Kudziwitsa omwe ndinakumana nawo ndisanayezedwe
    - Zina, tchulani ________

8. Kodi alipo wina mnyumba mwanu amene adapezeka ndi matenda a COVID-19?
    - Inde
    - Ayi

9. Kodi mwapeza kukhala panokha kunyumba, mutapezeka ndi matenda a COVID-19, kovuta?
    - Inde
    - Ayi

10. Ngati Inde pafunso lapitali, mungandiuzeko zovuta zomwe mwakumana nazo? _________

11. Kodi muli ndi malingaliro aliwonse amomwe tingathandizire kukonza kukhala kwanokha chifukwa chopezeka ndi matenda a COVID-19 kuti tichepetse kufala kwa matendawa?
    - Atha kulankhula zakukhosi mopanda malire

Zambia Questionnaires

1. *Information Sheet and consent form for Healthcare Worker Questionnaires*

Title of the proposed study: An Evaluation of Community-based Delivery and Administration of SARS-CoV-2 Antigen Rapid Tests

Investigators:

Background Purpose of the study:

This project led by the Ministry of Health (MOH) and Clinton Health Access Initiative (CHAI) aims to gather information on the implementation of a program involving point-of-care (POC) antigen testing for SARS-CoV-2, the virus causing the disease COVID-19. You are being invited to participate in a questionnaire because we are interested in the opinions and experiences of staff involved in POC antigen testing at this site.

Procedures:

You will be asked about your experience with the implementation of the POC COVID testing program at this site, the benefits, and challenges to the program, and the suggestions you have for improvements. The questionnaire will be read to you by a data collector and will last approximately 30-60 minutes during your working period

Risks/Discomforts:

Your position and your relationship with the MOH and CHAI will not be affected by whether or not you decide to participate in this questionnaire or by your responses. Your name and other personal information will not be requested and your participation in this study will be confidential.

Benefits:

Participation in this study may help to improve service delivery for people who wish to be tested for SARS CoV-2.

Compensation:

You will not be compensated for this questionnaire.

Questions:

Any individual who has complaints or questions about this study should contact:

Local PI name and contact information

Questions about participant rights:

For questions regarding your rights as a research participant contact:

Local PI name and contact information

Statement of voluntariness:

Participation in this questionnaire is completely voluntary. You may stop the questionnaire at any time to ask questions about participation or stop your participation completely. You can withdraw from the questionnaire at any point with no impact on your employment or performance. In the event that you withdraw your data and responses will be discarded and not kept on record.

Confidentiality:

Care will be taken to provide a private place to complete the questionnaire so that you feel free to speak. Questionnaire responses will be accessible only to the researchers and will not contain identifiable information. Specific information about you will never appear in a report or be published. Data collected will be stored on a secure, password-protected server and computer and only available to authorized CHAI staff. Your answers will be retained for three years after the end of the research project, or for a longer period if required by the local policy. Data from this study will be owned by each country's government and will be solely used to answer research questions around COVID-related policy.

STATEMENT OF CONSENT

I have been invited to participate in an evaluation of the operational assessment of the SARS-CoV-2 Antigen testing program. I have been selected to participate because I am a healthcare worker at this testing site. Participation will consist of a questionnaire that will last approximately 30-60 minutes. I understand that participation in this questionnaire is completely voluntary, and no compensation will be provided.

I have read the information about this study, or it has been read to me. I have had the opportunity to ask questions about it and any questions I have asked have been answered to my satisfaction. For any further questions, I may contact XXXXXXXXXXXXXXXXX on +26XXXXX. I voluntarily consent to be a participant in this evaluation, and I understand that I can stop at any time or choose not to answer any questions.

Name………………………………………..

Signature of participant ………………………………………………….

Date (DD/MM/YY) ………………………………………………

Name………………………………………………..

Signature of Interviewer ………………………………………………….

Date (DD/MM/YY) ………………………………………………

2. *Healthcare Worker questionnaire*

Date: ______________________________

Site: ____________________________

Gender: ____________________________

Age: _______________________________

Instructions: The format of this data collection is a semi-structured questionnaire to be read aloud to the respondent and responses entered electronically onto Android tablets. Everyone will be invited and at minimum, **one questionnaire should be conducted with the person responsible for providing COVID-19 Antigen testing at the site** (or the acting in-charge, if the in-charge is not available). Administration of the questionnaire should take place in a private room so that staff feel comfortable answering freely and honestly. Questionnaires are meant to last approximately 30-60 minutes. Questionnaires refer to the COVID-19 Antigen testing.

1. What is your role at the testing site?
   - o In-charge of site
   - o Nurse
   - o Lab technician
   - o Other, specify:

2. Have you received Training on COVID-19 antigen testing?
   - o Yes
   - o No

3. If yes, indicate how were you trained?
   - o Official training
   - o Unofficially trained by a peer
   - o Other (Explain): _____________

~~Do you provide COVID-19 testing at the site?~~

   - ~~o Yes~~
   - ~~o No~~

4. Have there been any patients who should have received COVID-19 Antigen tests who did not receive them?

- No
- Yes. Specify why the patient did not receive the test:
  - Too many patients so unable to provide care
  - Not enough test kits
  - Not enough staff
  - Other

5. In the last month, how long did **most** patients have to wait for their COVID-19 Antigen test results?
   - Less than 1 hour
   - 1-2 hours
   - 2-4 hours
   - 4-8 hours
   - 8-24 hours
   - 24 hours-1 week
   - More than 1 week

6. In the last week, did you have patients that have to wait for longer than 2 hours for their COVID-19 Antigen test results?
   - N0 (Skip to Q11)
   - Yes

7. In the last month, how often did the patients wait longer than 2 hours for their COVID-19 antigen test results?
   - Less than 1 out of every 10 patients
   - 1 to 3 out of every 10 patients
   - 4-6 out of every 10 patients
   - 7-10 out of every 10 patients
   - Others, specify

8. Have any patients expressed concerns about whether they can trust the results of the Antigen test because the results are available so rapidly?
   - No (Skip to Q13)
   - Yes, a few
   - Yes, many

9. Can you tell me more about the concerns of those patients? Note: Describe the comments made by the patients)
   - [_________]

10. Have site staff expressed concerns about whether they can trust the results?

- None (Skip to Q15)
- A few
- Many

11. Can you tell me more about the concerns of those staff members?
    - [__________]

Prompt: Now I would like to ask you questions pertaining to how often the following issues in the last month resulted in delays in returning test results?

12. High volume of COVID-19 Antigen tests requested at the site
    - Never
    - 1 time
    - 2-4 times
    - 4+ times

13. Limited number of trained operators to collect samples
    - Never
    - 1 time
    - 2-4 times
    - 4+ times

14. Limited number of trained testers to run the tests
    - Never
    - 1 time
    - 2-4 times
    - 4+ times

15. Stockouts of COVID-19 Antigen test kits
    - Never (Skip to Q21)
    - 1 time
    - 2-4 times
    - 4+ times

16. What was the average duration of the stockout?
    - Less than 1 day
    - 1 day to less than 1 week
    - 1 week to less than 1 month
    - 1 month or greater

17. How was the stockout fixed?
    - Waited until next scheduled shipment arrived

- Partner provided extra shipment to fill the gap in stock
- Other, specify:

18. Stockouts of PPE and other safety materials
    - Never (Skip to Q24)
    - 1 time
    - 2-4 times
    - 4+ times

19. What was the average duration of the stockout?
    - Less than 1 day
    - 1 day to less than 1 week
    - 1 week to less than 1 month
    - 1 month or greater

20. How was the stockout fixed?
    - Waited until next scheduled shipment arrived
    - Partner provided extra shipment to fill the gap in stock
    - Other, specify:

21. Other reasons for COVID-19 Antigen test processing delays: (specify)
    - Never
    - 1 time
    - 2-4 times
    - 4+ times

22. What in your experience has been going well with regards to Rapid Antigen testing at this site?
    - [__________]

23. What if any, factors are limiting the success of Rapid Antigen testing at this site?
    - [__________]

24. Do you have any ideas on how to make the Antigen testing program stronger at this site?
    - [__________]

3. *Information Sheet and consent form for individual questionnaires*

Title of the proposed study: An Evaluation of Community-based Delivery and Administration of SARS-CoV-2 Antigen Rapid Tests

Instructions for study staff: Please ask the participant to read this form carefully in either English or the local language based on their preference. If a participant cannot read or have trouble understanding, please read the information sheet to the participants in their preferred language.

Instructions for participants: Please read this form carefully or ask study staff to read the form to you. If you have any questions, please ask us. If you decide to participate in this research, you will be asked to sign this form. A copy of the signed form will be provided to you for your record.

Background & Purpose of the study: You are being invited to participate in a research study led by the Ministry of Health (MOH) and Clinton Health Access Initiative (CHAI) that is looking at ways to improve the SARS-CoV-2 Antigen testing program. This testing informs if you are infected and if you and your family members and contacts would need to quarantine to limit the spread of the virus. Here we will be asking questions to understand your testing experience at the testing site.

How many people will take part? Questionnaires will be offered to 10-15 participants in each site who have received an Antigen test.

Procedures: You will be asked about your experience with receiving the antigen test at this site. The questionnaire will last approximately 5-10 minutes.

Potential Risks:

The risks associated with participating in the study are considered to be minimal. Your name and other personal information will not be requested and your participation in this study will be confidential. Participation in the survey will not in any way affect the availability of your test result.

Potential Benefits:

This study has the potential to benefit you and the broader community by providing information that can be used to improve Antigen COVID-19 testing service delivery.

Compensation:

You will not be compensated for this study.

Voluntary participation:

Participation in this study is completely voluntary.

You are free to withdraw from this study at any time and for any reason. Refusal from the study will not have any impact on your access to testing, care, and treatment.

Questions:

Any individual who has complaints or questions about this study should contact the Study Principle Investigator at ………………………

Confidentiality:

Care will be taken to provide a private place to complete the questionnaire so that you feel free to speak. Questionnaire responses will be accessible only to the researchers and will not contain identifiable information. Specific information about you will never appear in a report or be published. Data collected will be stored on a secure, password-protected server and computer and only available to authorized CHAI staff. Your answers will be retained for three years after the end of the research project, or for a longer period if required by the local policy. Data from this study will be owned by each country's government and will be solely used to answer research questions around COVID-related policy.

Statement of consent:

I have been invited to participate in a questionnaire to assess my experiences with receiving tests for COVID-19 with an antigen test. I have been selected to participate because I have received an antigen test at this testing site. Participation will consist of a questionnaire that will last approximately 5-10 minutes. I understand that participation in this questionnaire is completely voluntary, and no compensation will be provided.

I have read the information about this study, or it has been read to me. I have had the opportunity to ask questions about it and any questions I have asked have been answered to my satisfaction. For any further questions, I may contact the Study Principle Investigator on the number provided should I have any additional questions. I voluntarily consent to be a participant in this study, and I understand that I can stop at any time or choose not to answer any questions.

Name of Participant: ___________________________

Signature of Participant: ________________________

Date (DD/MM/YY): ____________________________

Name of Staff: ________________________________

Signature of Staff: _____________________________

Date (DD/MM/YY): ____________________________

If illiterate

A literate witness must sign (if possible, this person should be selected by the participant and should have no connection to the research team). Participants who are illiterate should include their thumbprint as well.

I have witnessed the accurate reading of the consent form to the potential participant, and the individual has had the opportunity to ask questions. I confirm that the individual has given consent freely.

Print name of witness: ______________________ **AND** **Thumb print of participant**

Signature of witness: _______________________

Date (DD/MM/YY): _________________________

4. *Individual questionnaire*

Date: ________________________________

Testing site: _______________________________

Antigen test used: _______________________

Test Result: ______________________________

Gender: _________________________________

Age: ____________________________________

Symptoms: _______________________________

Instructions: The format of this data collection is a structured questionnaire to be read aloud to the respondent and responses entered electronically onto Android tablets. Questionnaire should be conducted with the individual receiving a COVID-19 Antigen testing. The questionnaire should take place in a private room so that individual feels comfortable answering freely and honestly. Questionnaires are meant to last approximately 5-10 minutes.

1. Before today, have you previously been tested for COVID-19?
   - Yes
   - No (Skip to Q6)

2. When did you receive your last test?
   - ______________(DD/MM/YYY)

3. Where did you get tested for COVID-19?
   - At the quarantine zone
   - At the hospital
   - At a MoH COVID -19 testing site
   - At border post
   - At the airport
   - At a community testing site
   - Other, specify _____

4. What was the result?
   - Positive
   - Negative
   - Invalid/Indeterminate
   - I don’t know/Prefer not to say

5. Which testing method was used?
   - Don’t know/Prefer not to say
   - RT-PCR
   - Antibody test
   - Antigen test

- Other, specify

6. How did you hear about COVID-19 testing was being offered at this site?
    - From HCW at the site
    - From local healthcare facility or hospital
    - From the announcement (newspaper, radio)
    - From a poster at the entrance to the site
    - From a friend
    - Other, specify
    - No response

7. Why did you choose to participate in testing today?
    - Had some symptoms
    - Curiosity
    - Have recently been in contact with someone who tested positive
    - To take good care of my health
    - Other, specify ________________________

8. Did you find the sample collection experience acceptable?
    - Very acceptable
    - Acceptable
    - Neutral
    - Not acceptable Specify reason: ________________________

9. Did you find the overall testing experience acceptable?
    - Very acceptable
    - Acceptable
    - Neutral
    - Not acceptable Specify reason: ________________________

10. After your sample was taken today, how long did it take to receive your COVID-19 test result?
    - Same day: less than 1 hours
    - Same day: 1 -2 hours
    - Same day: more than 2 hours
    - Was told to come back the next day
    - Never received

11. What's the maximum length of time that you desire for a COVID-19 test result to be received?
    - Same day: less than 1 hours

- Same day: 1 to 2 hours
- Same day: more than 2 hours
- Next Day
- Within 1 week
- At the next appointment (14 days)
- Other, specify___________

12. Do you trust the COVID-19 test result?
    - Yes
    - No, Specify: ____________________________

13. If you receive a positive test result today, how likely is it that you will quarantine for the recommended 14 days? Please feel free to specify your reason
    - Highly unlikely
    - Unlikely
    - Neither likely not unlikely
    - Likely
    - Highly likely
        - Reason for answer ____________________________
    - Decline to answer

14. Would you consider this COVID test in the future when you need to be screened for COVID-19?
    - Yes
    - No, specify: _____________________________

15. What do you think worked well about the COVID services you received today?
    - Open ended: _________________________________

16. Overall, would you recommend COVID testing at this testing site to your family and friends?
    - Yes
    - No, specify: __________________________________

17. Do you have any suggestions on how we can improve the testing service?
    - Open ended: ________________________________

5. *Information Sheet and consent form for post-test follow up call for individuals who test positive*

Title of the proposed study: Evaluation of Community-based Delivery and Administration of SARS-CoV-2 Antigen Rapid Tests in Zambia

Instructions for study staff: Please ask the participant to read this form carefully in either English or the local language based on their preference. If a participant cannot read or have trouble understanding, please read the information sheet to the participants in their preferred language.

Instructions for participants: Please read this form carefully or ask study staff to read the form to you. If you have any questions, please ask us. If you decide to participate in this part of the research, you will be asked to sign this form and provide a phone number that we can contact you on. A copy of the signed form will be provided to you for your record.

Background & Purpose of the study: You are being invited to participate in a research study led by the Ministry of Health (MOH) and Clinton Health Access Initiative (CHAI) that is looking at ways to improve the SARS-CoV-2 Antigen testing program. This testing informs if you have SARS-CoV-2 and if you and your family members and contacts would need to quarantine to limit the spread of the virus. Here we will be asking questions to understand your experience with quarantine after receiving a positive antigen result at the end of the required 14-day period.

Who will take part? Follow up calls at day 14 after receiving the positive result will be offered to **all participants in each site who have received a positive antigen test.**

Procedures: You will be asked over the phone about your experience with quarantine after receiving the antigen test at this site. The phone call will last approximately 5-10 minutes.

Potential Risks:

This study has the potential to benefit you and the broader community by providing information that can be used to improve antigen COVID-19 testing service delivery and COVID-19 surveillance and prevention measures.

Potential Benefits:

This study has the potential to benefit you and the broader community by providing information that can be used to improve antigen COVID-19 testing service delivery and COVID-19 surveillance and prevention measures.

Compensation:

You will not be compensated for this follow up call.

Voluntary participation:

Participation in this Post-test survey follow up call is voluntary. You are free to withdraw from call at any time and for any reason.

Refusal to receive the post-test follow up call will not have any impact on your access to testing, care, and treatment in the future

STATEMENT OF CONSENT

I have been invited to participate in an evaluation of the operational assessment of the SARS-CoV-2 Antigen testing study. I have been selected to participate because I received an antigen test at this testing site. Participation will consist of a follow up post-test survey call after the designated 14 days of quarantine. The phone call will last approximately 5-10 minutes. I understand that participation in this questionnaire is completely voluntary, and no compensation will be provided.

I have read the information about this study, or it has been read to me. I have had the opportunity to ask questions about it and any questions I have asked have been answered to my satisfaction. For any further questions, I may contact the study investigators. I voluntarily consent to be a participant in this evaluation, and I understand that I can stop at any time or choose not to answer any questions.

Name of Participant: ________________________ **Name of Staff:** _____________________________

Signature of Participant: _____________________ **Signature of Staff:** __________________________

Date (DD/MM/YY): ________________________ **Date (DD/MM/YY):** ________________________

Phone number to contact: ____

Indicate best time for a follow-up call: ______________________________

If illiterate

A literate witness must sign (if possible, this person should be selected by the participant and should have no connection to the research team). Participants who are illiterate should include their thumbprint as well.

I have witnessed the accurate reading of the consent form to the potential participant, and the individual has had the opportunity to ask questions. I confirm that the individual has given consent freely.

Print name of witness: _____________________ **AND** **Thumb print of participant**

Signature of witness: ______________________

Date (DD/MM/YY): ________________________

6. Post Test survey

Date : ________________________________

Gender: ______________________________

Age: _________________________________

Symptoms: ____________________________

Instructions: The call is a quick follow-up survey on the COVID-19 test you received at the Lusaka market. The information collected will be confidential and anonymous for operational research purposes.

1. Were you able to complete quarantine yourself for the recommended 10 days?
   - Yes
   - No
   - Prefer not to say

2. How many days total did you stay isolated?

3. Can you please share your reasons for not completing the quarantine?

4. Can you please share challenges with quarantine?

5. Did you take any other preventative actions during the first 10 days after your positive test?
   - Wearing mask
   - Working from home
   - Informed Contacts
   - Other, specify ________

6. Did anyone else in your family develop symptoms for COVID?
   - Yes
   - No
7. If yes, how many
8. If yes, how many tested positive?
